# Maternal DHA-rich n-3 PUFAs supplementation interacts with *FADS* genotypes to influence the profiles of PUFAs in the colostrum among Chinese Han population: a birth cohort study

**DOI:** 10.1186/s12986-022-00683-3

**Published:** 2022-07-23

**Authors:** Ping Li, Yuhui Chen, Jieyun Song, Lailai Yan, Tiantian Tang, Rui Wang, Xiuqin Fan, Yurong Zhao, Kemin Qi

**Affiliations:** 1 Laboratory of Nutrition and Development, Key Laboratory of Major Diseases in Children, Ministry of Education, Beijing Pediatric Research Institution, Beijing Children’s Hospital, Capital Medical University, National Center for Children’s Health, No.56 Nan-li-shi Road, Xicheng District, Beijing, 100045 China; 2 Department of Obstetrics and Gynecology, Fuxing Hospital, Capital Medical University, No. 20 Fu-xing-men-wai Street, Xicheng District, Beijing, 100038 China; 3 Institute of Child and Adolescent Health, Department of Child, Adolescent and Women’s Health, School of Public Health, Peking University, Beijing, 100191 China

**Keywords:** Single nucleotide polymorphisms, Matrix-assisted laser desorption ionization time of flight mass spectrometry, Polyunsaturated fatty acids, DHA supplementation, Colostrum, Birth cohort

## Abstract

**Background:**

The single nucleotide polymorphisms (SNPs) in the fatty acid desaturases and elongases might associate with the endogenous synthesis of polyunsaturated fatty acids (PUFAs). However, the related epidemiological evidence is still conflicting. So we aimed to clearly evaluate the interactions between maternal DHA-rich n-3 PUFAs supplementation and the known 26 SNPs on the profiles of PUFAs in the colostrum using a Chinese birth cohort.

**Methods:**

Totally, 1050 healthy mother-infant pairs were enrolled in this study at gestational 6–8 weeks when they established their pregnancy files at Fuxing Hospital affiliated to Capital Medical University in Beijing from January to December 2018. Meanwhile, their venous blood samples were obtained for DNA extraction to detect the genotypes of SNPs in the *Fads1, Fads2, Fads3, Elovl2* and *Elovl5* using the Matrix-Assisted Laser Desorption Ionization Time of Flight Mass Spectrometry. Then the colostrum samples were collected to determine the profiles of PUFAs by gas chromatography.

**Results:**

Maternal DHA-rich n-3 PUFAs supplementation from the early and middle pregnancy could reduce the infant BMI at birth, and impact the profiles of PUFAs in the colostrum, as higher n-3 PUFAs (EPA, DHA, DHA/ALA and DHA/EPA), lower n-6 PUFAs (AA and AA/LA) and ∑-6/n-3ΣPUFAs. Moreover, there were significant correlations between multiple SNPs and the profiles of n-6 PUFAs (rs76996928 for LA, rs174550, rs174553 and rs174609 for AA, rs174550 and rs76996928 for AA/LA) and n-3 PUFAs in the colostrum (rs174448, rs174537, rs174550, rs174553, rs174598, rs3168072, rs174455 and rs174464 for ALA, rs174550, rs174553 and rs174598 for EPA, rs174455 and rs174464 for DHA, rs174448 and rs3168072 for DHA/EPA) using the multiple linear regressions by adjusting the maternal age, gestational week, mode of delivery, infant sex and BMI at birth, and all these above significant SNPs had the cumulative effects on the profiles of PUFAs. Furthermore, the pairwise comparisons also showed the meaningful interactions between maternal DHA-rich n-3 PUFAs supplementation and related genotypes of SNPs (rs76996928 for LA, rs174598 for EPA, rs174448 for DHA and DHA/EPA) on the contents of PUFAs in the colostrum.

**Conclusions:**

Results from this birth cohort study proved that the pregnant women with the following SNPs such as *Fads3* rs174455 T, *Fads3* rs174464 A and *Fads1* rs174448 G alleles should pay more attention on their exogenous DHA supplementation from the early and middle pregnancy for the blocked endogenous synthesis.

*Trial registration*: This study was approved by the Ethics Committee of Beijing Pediatric Research Institution, Beijing Children’s Hospital affiliated to Capital Medical University (2016–08), which was also registered at the website of http://www.chictr.org.cn/showproj.aspx?proj=4673 (No: ChiCTR-OCH-14004900).

**Supplementary Information:**

The online version contains supplementary material available at 10.1186/s12986-022-00683-3.

## Background

Recently, mounting evidence has addressed that n-6 and n-3 polyunsaturated fatty acids (PUFAs), especially the docosahexaenoic acid (DHA), play a significant role on the early neurodevelopment, cognitive development, and fetal growth by regulating the related genes in the cell signals, cell fluidity, oxidative stress, glucose metabolism, adipogenic proliferation and multi-differentiation of stem cells through the epigenetic pathways [1, 2]. Meanwhile, there are many compelling epidemiological and animal researches, which also have confirmed that both maternal n-3 PUFAs deficiency and excess could have long-lasting adverse influences on the health in early infancy and/or even the adulthood in later life [3, 4]. And during this sensitively pregnant stage, the umbilical cord and placenta are the fetal sole sources of all important nutrients from their mothers, by which maternal n-3 PUFAs intake and its metabolites during the pregnancy can determine the availability of essential DHA for their offspring during the first months of life [5]. Hence, the adequate maternal n-3 PUFAs (especially DHA) intake is critical for their fetus life onward [6]. However, the results from many well-designed randomized controlled trials have proved that prenatal DHA intake is yielded insufficient, so it is very urgent and important for improving the fitness of their infants through giving individualized appropriate DHA intake during the pregnancy [7].

So far, increasing epidemiological evidence implicates that the profiles of maternal n-6 and/or n-3 PUFAs, mainly including the linoleic acid (LA), α-linolenic acid (ALA), arachidonic acid (AA), eicosapentaenoic acid (EPA) and DHA, are depended on both the exogenous intake and endogenous synthesis. As the previous studies, the exogenous consumption of n-3 PUFAs are mainly from the deep-sea fish (yellow croaker, codfish, trichiurus haumela, sardine, sockeye salmon, tynny, saury, muraenesox, cinereus and braised flatfish head), various rapeseed oils and exogenous DHA-rich n-3 PUFAs supplementation on average [5, 6]. However, according to the Chinese dietary structure and limited exogenous DHA-rich n-3 PUFAs supplementation, it will inevitably lead to the lack of exogenous n-3 PUFAs intake during the pregnancy [7, 8]. In this condition, the endogenous synthesis of n-3 PUFAs (especially the EPA and DHA) has become a very important source using many clinical and epidemiological studies, in which their biosynthesis are self-synthesis catalyzed by many enzymes, mainly the fatty acid desaturases (FADS) and elongases (ELOVL).

Recently, the previous studies have proved that the enzymes of FADS and ELOVL are respectively encoded by the genes such as *Fads1*, *Fads2*, *Fads3*, *Elovl2* and *Elovl5.* And their expressions can be regulated by many single-nucleotide polymorphisms (SNPs) to further impact the profiles of n-6 PUFAs (LA can be converted to AA) and n-3 PUFAs (ALA can be converted to EPA, DHA and etc.) [2, 8–14]. However, it is still inconsistent of the interactions between the known 26 SNPs in the *Fads1*, *Fads2*, *Fads3*, *Elovl2* and *Elovl5,* and exogenous DHA intake on the profiles of circulating PUFAs in lots of researches [15–17]. Moreover, little is still known about how the joint associations between gene–gene or/and gene-environment elements impact the profiles of n-6 and n-3 PUFAs in the colostrum. Furthermore, it has been a call to include the genetic assessments of SNPs in the cohort to discuss the possible interactions across the studies on the basis of these important genetic variations and exogenous DHA intake on the profiles of PUFAs in different populations. So understanding the above interactions may help the dietary modifications to realize the individualized DHA-rich n-3 PUFAs supplementation among the pregnant women to ensure the fitness of their infants. What is more, our previous study also had proved that there were positive correlations between the profiles of PUFAs in the colostrum and those in the umbilical cord blood to some extent [18], so the colostrum could be chosen as the target biological for their sampling convenience. Therefore, we addressed this gap by describing the associations and interactions between maternal DHA-rich n-3 PUFAs supplementation, and the known 26 SNPs in the *Fads1, Fads2, Fads3, Elvol2* and *Elvol5* on the profiles of n-6 and n-3 PUFAs in the colostrum (particularly LA, AA, ALA, EPA and DHA) among the Chinese Han population using a birth cohort study.

## Methods

### Study design and participants

This study was based on a prospective birth cohort among Chinese Han population, which was carried out at Fuxing Hospital affiliated to Capital Medical University in Beijing, China. In this cohort, 1050 healthy mother-infant pairs were recruited from Jan 2018 to Dec 2018 at gestational 6–8 weeks when they established their pregnancy files according to the stringent inclusion and exclusion criteria. Specifically, the eligible women were healthy at 20–40 years old and intended to deliver in this hospital without smoking and drinking history. Conversely, the pregnant women with gestational hypertension, hypothyroidism, diabetes, heart diseases, hepatitis, cirrhosis, severe fatty liver, nephritis, severe anemia, leukemia, malignant tumor of immune system and hemorrhagic diseases, medical history of using antibiotics and thyroxine related drugs, the individuals without colostrum after delivery, and their infants with birth defects, genetic and metabolic diseases, ischemia, and hypoxia were all excluded from this study. Besides, the participants who were intermittently given exogenous DHA-rich n-3 PUFAs supplementation were also rejected to ensure the consistency of the data.

This study was approved by the Ethics Committee of Beijing Pediatric Research Institution, Beijing Children's Hospital affiliated to Capital Medical University (No: 2016–08), which was also registered at the website of http://www.chictr.org.cn/show proj.aspx?proj = 4673 (No: ChiCTR-OCH-14004900). Meanwhile, all subjects were granted both the written informed consents and Health Insurance Portability and Accountability Act Authorization after they were clearly informed the significance of this survey by the trained investigators.

### Basic information and food frequency questionnaires

The basic information questionnaires, including maternal age, height, pre-pregnancy weight, pre-pregnancy body mass index (BMI), prenatal weight, prenatal BMI, gestational age, mode of delivery and infant sex, were respectively performed among the participants by the trained investigators through face to face method when they were involved in this study. Meanwhile, the anthropometric measurements (lenght and weight) were collected at birth by the pediatricians using the nearest millimeter (HW-1000HW-2000, China) on a digital board with three repeated samples to calculate the mean values when they were delivered. Then the head circumference was measured by the tapes.

The food frequency questionnaires (FFQ) were used to determine the total DHA intake both from the dietary and exogenous DHA-rich n-3 PUFAs supplementation at gestational 12 (early pregnancy), 27 (middle pregnancy) and 36 week (late pregnancy). Exactly, the pregnant women were asked to obtain the dietary specified averagely amounts and types of foods with DHA-rich n-3 PUFAs, which were mainly included the deep-sea fish (yellow croaker, codfish, trichiurus haumela, sardine, sockeye salmon, tynny, saury, muraenesox, cinereus and braised flatfish head), algae, nuts, egg yolk and various seed oils (flax seed oil, peril-la seed oil and walnut oil). In this process, nine possible frequency categories were ranged from never/almost never to ≥ 6 times per week to calculate the total dietary DHA intake using the SY-2 Nutrition Analysis Software. Meanwhile, the open-ended questions were asked to collect and calculate the exogenous DHA-rich n-3 PUFAs supplementation, including their usual time, brands, doses and types according to the commercial specifications. Therefore, total DHA intake during the pregnancy was the sum from the dietary and exogenous DHA-rich n-3 PUFAs supplementation. Then, the subjects were respectively divided into four groups according to the firstly gestational weeks of exogenous DHA-rich n-3 PUFAs supplementation, which were exogenous DHA-rich n-3 PUFAs supplementation groups from the early (0–12 week, S1 group), middle (13–27 week, S2 group) and late pregnancy (> 27 week, S3 group), with non-exogenous DHA-rich n-3 PUFAs supplementation during the whole pregnancy as the control group.

### Blood and colostrum collection

1 mL venous blood was obtained from the pregnant women for DNA extraction when they were recruited into this study. Meanwhile, they were asked to collect 5 mL colostrum within postpartum 3–5 days. All biological samples were immediately transported to the laboratory using the dry ice and stored at -80℃ refrigerator until the usage.

### Profiles of PUFAs in the colostrum

The profiles of PUFAs were measured by the gas chromatography (GC) after they were extracted from 100 μL colostrum with 2 mL mixture of chloroform and methanol (1:9), containing 0.001% butylated hydroxytoluene (BHT) and 2μL pentadecanoic acid standard (C15:0, cat. no. P6125, Sigma-Aldrich Chemie GmbH, Germany, 0.15 g/mL ethanol). The above mixture was heated at 100 °C for 1 h, while 5 mL 6% K_2_CO_3_ solution and 200 μL n-hexane were added to these tubes, mixed on a vortex, and centrifuged at 3000 rpm/min for 15 min (10℃). Then the clear n-hexane top layer was transferred to the GC auto sampler vials to determine the profiles of PUFAs using the Agilent 6890 N GC with the flame ionization detection (P/N 19091J-433, HP-5 capillary column was 30 m × 0.32 mm × 0.25 μm). All measurements were done in the duplicate and reported as the averages, in which the profiles of PUFAs were expressed as the proportions of fatty acid/all fatty acids.

### DNA extraction from the venous blood

The whole DNA was extracted from 200 mL venous blood using the DNA extraction kits according to manufacturer’s instructions (cat. no. DP348-03, Tiangen Biotech (Beijing) Co. Ltd, China). The quantity and quality of the purified DNA samples were respectively evaluated by the ND-1000 spectrophotometer (NanoDrop 2000C, Thermo Fisher Scientific, CN) and agarose gel electrophoresis.

### Selection of the SNPs and their genotypes

All 26 SNPs were selected in the genes of *Fads1, Fads2, Fads3, Elvol2* and *Elvol5,* which were sited at the potential 5′ or 3′ regulatory regions in the strong linkage disequilibrium (LD) to enable their genotypes (*r*^2^ > 0.7) [9, 19–30]. As shown in the Additional file 1: Tables S1, S2, the genotypes of rs174448 (A/G, 11:61,872,101), rs174537 (G/T, 11:61,785,208), rs174550 (T/C, 11:61,804,006) and rs174553 (G/A, 11:61,807,686) in the *Fads1*, rs174598 (A/T, 11:61,853,722), rs174602 (C/T, 11:61,856,942), rs174609 (T/C, 11:61,860,339), rs174619 (G/A, 11:61,862,194), rs498793 (C/T, 11:61,857,233) and rs3168072 (A/T, 11:61,864,038) in the *Fads2*, rs174455 (C/T, 11:61,888,710), rs174464 (G/A, 11:61,890,454) and rs76996928 (T/C, 11:61,883,454) in the *Fads3*, rs1323739 (G/C, 6:11,004,328), rs2295602 (C/T, 6:11,005,609), rs2180725 (T/C, 6:11,025,187), rs3798710 (G/C, 6:11,002,550) and rs78793420 (T/C, 6:11,039,168) in the *Elvol2*, and rs209512 (A/G, 6:53,338,779), rs2281274 (T/C, 6:53,278,756), rs2294852 (C/G, 6:53,292,416), rs2397142 (C/G, 6:53,335,501), rs6909592 (G/C, 6:53,298,292), rs9349665 (T/C, 6:53,341,701), rs9395858 (C/T, 6:53,313,689) and rs12207094 (A/T, 6:53,339,377) in the *Elvol5* were associated with the strongest GWAS signals for up to 20% of their variations using the International HapMap Project SNP database and NCBI database (http://www.ncbi. nlm.nih.gov/snp).

All above 26 SNPs were measured by the Matrix-Assisted Laser Desorption Ionization Time of Flight Mass Spectrometry (MALDI-TOF-MS, Sequenom) with the validated primers in the Additional file 1: Table S1, in which the genotyping success rate was more than 97% and respectively presented in the Chromosome 11 (*Fads1, Fads2* and *Fads3*) and 6 (*Elovl2* and *Elovl5*). Their minor allele frequencies (MAF) were ranged from 0.138 to 0.441. Then their genotype analysis was performed using the graphical Java interface of THESIAS software package (http://ecgene.net/ genecanvas). Meanwhile, the program was based on the maximum likelihood model linked to the SEM algorithm using the statistically reconstruct genotypes in the unrelated individuals and then investigated the effects of covariate adjusted genotypes as well as their interactions between maternal DHA-rich n-3 PUFAs supplementation and the related significant genotypes of 26 SNPs on the profiles of PUFAs among all 1050 participants according to the Hapmap database (http://hapmap.ncbi.nlm.nih.gov).

### Statistical analysis

The statistical analysis was carried out using the SPSS 21.0, in which *P* < 0.05 was recognized as the significant importance. Exactly, the normal distribution of outcome variables was evaluated both by the Kolmogorov–Smirnov test and distributional Q-Q plot. All data was expressed as mean (geometric mean) ± standard error (SE) and mean changes with respect to the baseline. The differences of the general characteristics were detested with one-way analysis of variance (ANOVA) and χ^2^ test. The distributions of all 26 SNPs were determined for the deviation from Hardy–Weinberg equilibrium using the χ2 test. Inter-locus LD based on the observed numbers of the genotypes in the *Fads1, Fads2, Fads3, Elvol2* and *Elvol5* was established using the CubeX software (http://www.oege.org/software/cubex/). Then the genetic data was analyzed for each SNP separately and categorized as the homozygous for the major allele by changing to the numerical variables. Meanwhile, the linear regression was used to assess the known 26 SNPs and maternal DHA-rich n-3 PUFAs supplementation on the profiles of PUFAs. Furthermore, the interactions between maternal DHA-rich n-3 PUFAs supplementation and the differently related genotypes of SNPs on the profiles of PUFAs were explored using the partial regression analysis by adjusting the maternal age, gestational week, mode of delivery, infant sex and BMI at birth.

## Results

### Basic characteristics of the subjects in this study

**Fig. 1:**
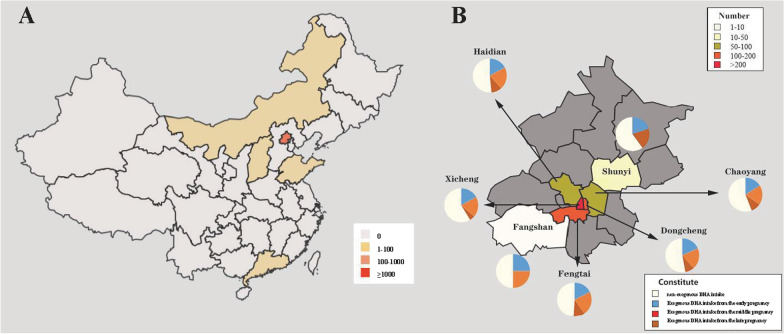
Characteristics of maternal exogenous DHA-rich n-3 PUFAs supplementation and geographical distributions of the subjects in this birth cohort.** A** showed the geographical distributions.** B** demonstrated maternal exogenous DHA-rich n-3 PUFAs supplementation in different districts of Beijing. *Note*: PUFAs: polyunsaturated fatty acids

The regional distribution of the 1076 Chinese Han participants (2 from Mongolia Autonomous Region, 5 from Shanxi province, 5 from Shandong province, 4 from Guangzhou province and 1050 from Beijing) in this study was shown in Fig. 1A. Then 1050 mother-infant pairs in Beijing were evolved for the following-up analysis (Fig. 1B), in which 679 subjects were from Xicheng (64.67%), 97 from Dongcheng (9.24%), 96 from Fengtai (9.14%), 93 from Haidian (8.86%), 71 from Chaoyang (6.76%), 5 from Fangshan (0.47%) and 9 from Shunyi Districts (0.86%). As shown in Table 1, daily DHA intake was the sum from the dietary and exogenous DHA-rich n-3 PUFAs supplementation. Exactly, the dietary DHA intake (162.02 ± 25.96 mg/day) was less than its recommendation, which were not significantly different among the S1, S2, S3 and control groups (*P* > 0.05). Meanwhile, the exogenous DHA intake were also similar among the S1 (324.51 ± 20.60 mg/day), S2 (337.75 ± 46.99 mg/day) and S3 groups (338.20 ± 40.71 mg/day) (*P* > 0.05),so it was meaningful to discuss the effects of maternal DHA-rich n-3 PUFAs supplementation on the profiles of PUFAs in the colostrum according to the initially different gestation periods. Then, all subjects were divided into four groups, including the exogenous DHA-rich n-3 PUFAs supplementation groups from the early (S1 group, n = 172, 16.38%), middle (S2 group, n = 197, 18.76%) and late pregnancy (S3 group, n = 65, 6.19%), and non-exogenous DHA-rich n-3 PUFAs supplementation group (control group, n = 616, 58.67%). Meanwhile, the percents of maternal DHA-rich n-3 PUFAs supplementation during the whole pregnancy were coincident among different districts of Beijing such as Xicheng, Dongcheng, Fengtai, Haidian, Chaoyang, Fangshan and Shunyi districts (Fig. 1B, *P* > 0.05).

**Table 1:** Basic characteristics of the subjects in the study

Indicators	All subjects (n = 1050)	Exogenous DHA-rich n-3 PUFAs supplementation groups (n = 434)			Control group (n = 616)	*P*^&^
		S1 group (n = 172)	S2 group (n = 197)	S3 group (n = 65)		
*Pregnant woman*						
Age (year)	32.15 ± 4.24	32.58 ± 4.03	31.88 ± 3.99	33.18 ± 2.44	32.00 ± 4.57	0.735
Height (cm)	163.11 ± 4.72	162.33 ± 5.38	165.23 ± 5.35	163.18 ± 5.36	162.64 ± 4.26	0.053
Pre-pregnancy weight (kg)	58.39 ± 9.19	59.14 ± 9.79	60.09 ± 8.62	57.91 ± 11.11	58.38 ± 9.19	0.795
Pre-pregnancy BMI (kg/m^2^)	22.08 ± 3.22	22.40 ± 3.26	22.01 ± 3.04	21.68 ± 3.76	22.05 ± 3.21	0.906
Prenatal weight (kg)	73.16 ± 9.10	73.15 ± 9.22	75.25 ± 8.39	70.81 ± 9.06	72.74 ± 9.32	0.460
Prenatal BMI (kg/m^2^)	27.48 ± 3.15	27.73 ± 3.34	27.60 ± 3.16	26.55 ± 2.77	27.47 ± 3.13	0.749
Weight gain (kg)	14.37 ± 4.83	14.02 ± 4.40	15.17 ± 4.81	12.91 ± 3.36	14.36 ± 5.11	0.359
BMI gain (kg/m^2^)	5.41 ± 1.83	5.32 ± 1.70	5.59 ± 1.86	4.87 ± 1.37	5.43 ± 1.91	0.714
Gestational age (week)	39.08 ± 1.40	39.14 ± 1.38	39.03 ± 1.31	38.55 ± 0.82	39.13 ± 1.49	0.920
Mode of delivery (Natural, %)	736 (70.10)	108 (62.79)	137 (69.54)	42 (64.62)	449 (72.89)	0.142
Daily dietary DHA intake (mg/day)	162.02 ± 25.96	144.81 ± 20.43	165.25 ± 20.75	157.75 ± 25.41	166.23 ± 29.22	0.894
Exogenous DHA supplementation (mg/day)	–	324.51 ± 20.60	337.75 ± 46.99	338.20 ± 40.71	–	0.907
*Infant*						
Sex (Boy, %)	532 (50.67)	86 (50.00)	100 (50.76)	32 (49.23)	314 (50.97)	0.671
Length (cm)	49.52 ± 1.53	49.60 ± 2.18	50.04 ± 1.92	49.00 ± 1.52	49.38 ± 1.23	0.374
Weight (kg)	3.47 ± 0.43	3.38 ± 0.48	3.30 ± 0.47	3.39 ± 0.40	3.55 ± 0.41	0.055
BMI (kg/m^2^)	14.14 ± 0.91	13.74 ± 0.92*	13.18 ± 0.79*^#^	14.12 ± 0.48	14.56 ± 0.99	0.041
Head circumference (cm)	35.14 ± 1.37	35.21 ± 1.48	35.47 ± 1.12	35.00 ± 1.14	35.02 ± 1.44	0.408

*PUFAs* Polyunsaturated fatty acids, *BMI* Body mass index

*Compared with the control group, *P* < 0.05

^#^Compared with the S3 group, *P* < 0.05

^&^The quantitative data was analyzed by ANOVA (q test), while the qualitative data (mode of delivery and sex) was analyzed by χ^2^ test among all these four groups. S1 group: exogenous DHA-rich n-3 PUFAs supplementation at the early pregnancy (0–12 week), S2 group: exogenous DHA-rich n-3 PUFAs supplementation at the middle pregnancy (13–27 week), S3 group: exogenous DHA-rich n-3 PUFAs supplementation at the late pregnancy (> 27 week), Control group: non-exogenous DHA-rich n-3 PUFAs supplementation during the whole pregnancy

The other basic characteristics of all subjects (Table 1) was as following: the maternal age was (32.15 ± 4.24) years, their height was (163.11 ± 4.72) cm, the pre-pregnancy weight was (58.39 ± 9.19) kg, pre-pregnancy BMI was (22.08 ± 3.22) kg/m^2^, prenatal weight was (73.16 ± 9.10) kg, prenatal BMI was (27.48 ± 3.15) kg/m^2^, gestational age was (39.08 ± 1.40) weeks, and 70.10% infants were natural delivery, while the infants at birth were 50.67% boy, (49.52 ± 1.53) cm, (3.47 ± 0.43) kg, and (35.14 ± 1.37) cm (head circumference), which were not significantly different among the S1, S2, S3 and control groups (*P* > 0.05). However, the BMI values in both the S1 (13.74 ± 0.92 kg/m^2^) and S2 groups (13.18 ± 0.79 kg/m^2^) were lower than those in the S3 (14.12 ± 0.48 kg/m^2^) and control groups (14.56 ± 0.99 kg/m^2^).

### Distributions of the 26 SNPs among the participants in different groups

**Fig. 2:**
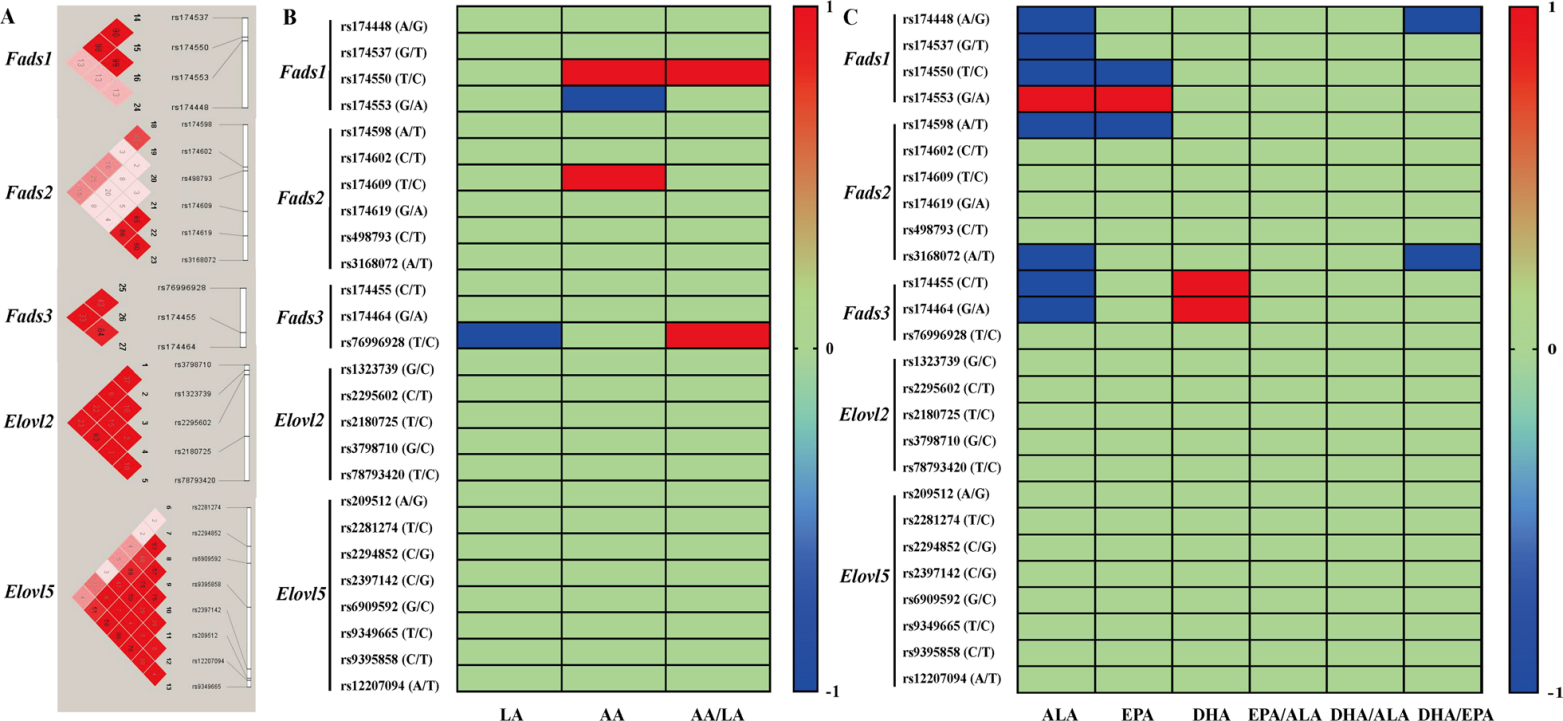
Effects of the 26 SNPs in the fatty acid desaturases and elongases on the profiles of PUFAs in the colostrum.** A** presented the linkage disequilibrium (LD) between the 26 SNPs in the *Fads1, Fads2, Fads3, Elvol2* and *Elvol5* and fatty acid synthesis (r^2^ × 100). **B **showed the profiles of n-6 PUFAs (LA, AA and AA/LA).** C** presented the profiles of n-3 PUFAs (ALA, EPA, DHA, EPA/ALA, DHA/ALA and DHA/EPA). Note: In the Fig. 2**B** and 2**C**, red, blue and green respectively demonstrated there were positive, negative and no significant associations between the 26 SNPs and the profiles of n-6 and n-3 PUFAs. *Fads* Fatty acid desaturases, *Elovl* Elongase of very long chain fatty acid, *PUFAs* Polyunsaturated fatty acids, *SNPs* Single nucleotide polymorphisms, *LA* Linoleic acid, *AA* Arachidonic acid, *ALA* α-Linolenic acid, *EPA* Eicosapentaenoic acid, *DHA* Docosahexaenoic acid

Table 2 and Additional file 1: Table S1 demonstrated the distributions of the 26 SNPs in the *Fads1, Fads2*, *Fads3*, *Elovl2* and *Elovl5* among the participants in different groups. The call rates of all 26 SNPs were more than 99%, the genotype accuracy which was assessed by the inclusion of duplicates in the array was more than 98%, and the negative control (water blank) was included in each plate. And the genotypes of these 26 SNPs did not deviate from the Hardy–Weinberg equilibrium among the S1, S2, S3 and control groups (Table 2 and Fig. 2A, *P* < 0.05).

**Table 2:** The distributions of 26 SNPs in the fatty acid desaturases and elongases of the subjects in this study

Gene	SNPs	Call rate (%)	Allele*	Ratio^#^	Genotypes	All subjects (n = 1050)	Exogenous DHA-rich n-3 PUFAs supplementation groups (n = 434)			Control group (n = 616)	*P*^&^
							S1 group (n = 172)	S2 group (n = 197)	S3 group (n = 65)		
*Fads1*	rs174448	99.94	A	0.810	AA/GG/AG	695/39/316	112/7/53	131/7/59	44/3/18	408/22/186	0.991
	rs174537	99.97	G	0.631	GG/TT/GT	419/141/490	68/24/80	79/24/94	26/9/30	246/84/286	0.988
	rs174550	99.91	T	0.619	CC/TT/CT	155/409/486	27/64/81	27/78/92	10/25/30	91/242/283	0.377
	rs174553	99.89	G	0.369	AA/GG/AG	424/152/474	68/26/78	81/27/89	26/10/29	249/89/278	0.830
*Fads2*	rs174598	99.18	A	0.361	AA/TT/AT	147/438/465	23/71/78	29/81/87	9/28/28	86/258/272	0.849
	rs174602	99.84	C	0.743	CC/TT/CT	67/576/407	10/95/67	12/108/77	5/36/24	40/337/239	0.940
	rs174609	99.89	T	0.824	CC/TT/CT	35/719/296	6/117/49	6/134/57	3/44/18	20/424/172	0.843
	rs174619	99.42	G	0.766	AA/GG/AG	62/625/363	11/103/58	12/117/68	3/40/22	36/365/215	0.972
	rs498793	100.00	C	0.921	CC/TT/CT	896/7/147	148/1/23	168/2/27	55/1/9	525/3/88	0.831
	rs3168072	99.91	A	0.836	AA/TT/AT	743/33/274	122/5/45	140/6/51	46/2/17	435/20/161	0.992
*Fads3*	rs174455	100.00	C	0.702	CC/TT/CT	521/93/436	84/17/71	98/16/83	34/5/26	305/55/256	0.082
	rs174464	99.84	G	0.785	AA/GG/AG	62/664/324	10/109/53	11/124/62	4/42/19	37/389/190	0.316
	rs76996928	99.67	T	0.541	CC/TT/CT	239/327/484	39/54/79	46/60/91	15/20/30	139/193/284	0.773
*Elovl2*	rs1323739	99.49	G	0.676	CC/GG/CG	99/471/480	15/78/79	19/87/91	6/30/29	59/276/281	0.226
	rs2295602	99.43	C	0.911	CC/TT/CT	878/11/116	145/2/25	165/2/30	54/1/10	514/6/96	0.996
	rs2180725	100.00	T	0.759	CC/TT/CT	66/613/371	12/97/63	12/116/69	4/38/23	38/362/216	0.983
	rs3798710	99.97	G	0.565	CC/GG/CG	205/344/501	32/58/82	39/64/94	13/21/31	121/201/294	0.939
	rs78793420	99.49	T	0.759	CC/TT/CT	55/603/392	9/97/66	10/114/73	4/38/23	32/354/230	0.937
*Elovl5*	rs209512	100.00	A	0.435	AA/GG/AG	197/331/522	32/53/87	38/61/98	12/21/32	115/196/305	1.000
	rs2281274	99.84	T	0.793	CC/TT/CT	40/660/350	7/109/56	7/125/65	3/41/21	23/385/208	0.919
	rs2294852	99.75	C	0.616	CC/GG/CG	401/154/495	64/26/82	76/29/92	26/9/30	235/90/291	0.997
	rs2397142	99.82	C	0.666	CC/GG/CG	479/127/444	79/20/73	90/23/84	30/8/27	280/76/260	0.908
	rs6909592	99.94	G	0.605	CC/GG/CG	168/391/491	28/65/79	31/73/93	10/25/30	99/228/289	0.924
	rs9349665	100.00	T	0.718	CC/TT/CT	91/550/409	14/91/67	17/103/77	6/35/24	54/321/241	0.776
	rs9395858	99.86	C	0.710	CC/TT/CT	532/87/431	87/15/70	101/15/81	33/5/27	311/52/253	0.505
	rs12207094	100.00	A	0.896	AA/TT/AT	845/9/196	139/2/31	158/2/37	52/1/12	496/4/116	0.774

*SNPs* Single nucleotide polymorphisms, *Fads* Fatty acid desaturases, *Elovl* Elongase of very long chain fatty acid, *PUFAs* Polyunsaturated fatty acids

*The effective alleles were determined according to the literature and the effect values which were positively associated between the genotypes of SNPs and contents of DHA in the colostrum

^#^The ratio was calculated by the effective alleles/not-effective alleles

^&^Data was analyzed by χ^2^ test among all these four groups. S1 group: exogenous DHA-rich n-3 PUFAs supplementation at the early pregnancy (0–12 week), S2 group: exogenous DHA-rich n-3 PUFAs supplementation at the middle pregnancy (13–27 week), S3 group: exogenous DHA-rich n-3 PUFAs supplementation at the late pregnancy (> 27 week), Control group: non-exogenous DHA-rich n-3 PUFAs supplementation during the whole pregnancy

### Effects of maternal exogenous DHA-rich n-3 PUFAs supplementation on the profiles of PUFAs in the colostrum

Based on the similar genotypes of 26 SNPs, it was discussed the effects of maternal exogenous DHA-rich n-3 PUFAs supplementation on the profiles of PUFAs in the colostrum. As shown in Table 3, the percentages of AA, AA/LA and n-6/n-3 ΣPUFAs were lower, with higher percents of n-3 PUFAs, EPA, DHA, DHA/ALA and DHA/EPA in the S1 and S2 groups than those in the S3 and control groups (*P* < 0.05).

**Table 3:** Effects of exogenous DHA-rich n-3 PUFAs supplementation during the pregnancy on the profiles of PUFAs in the colostrum

Indicators	Exogenous DHA-rich n-3 PUFAs supplementation groups (n = 434)			Control group (n = 616)	*P*^&^
	S1 group (n = 172)	S2 group (n = 197)	S3 group (n = 65)		
∑SFAs	37.83 ± 4.92	38.33 ± 5.24	39.14 ± 3.99	39.08 ± 5.41	0.451
C12:0	4.66 ± 2.89	4.92 ± 2.88	5.70 ± 2.53	6.11 ± 3.87	0.110
C14:0	4.46 ± 1.68	4.67 ± 1.57	4.57 ± 1.53	4.57 ± 1.68	0.932
C16:0	23.37 ± 2.30	23.32 ± 2.34	23.46 ± 1.31	22.96 ± 2.09	0.470
C18:0	5.34 ± 0.77	5.41 ± 0.79	5.39 ± 0.67	5.44 ± 0.91	0.911
∑MUFAs	38.51 ± 6.79	37.83 ± 8.29	39.02 ± 3.06	37.34 ± 3.92	0.679
C16:1	2.30 ± 0.69	2.38 ± 0.59	2.41 ± 0.61	2.24 ± 0.67	0.477
C18:1	36.21 ± 6.91	36.81 ± 4.51	36.61 ± 2.75	35.77 ± 6.30	0.705
n-6 PUFAs	22.05 ± 6.29	22.33 ± 5.62	20.18 ± 2.64	22.38 ± 6.80	0.325
C18:2n-6 (LA)	21.02 ± 6.79	21.25 ± 6.05	19.00 ± 2.76	21.05 ± 6.73	0.644
C20:4n-6 (AA)	0.65 ± 0.15*^#^	0.69 ± 0.16*^#^	0.76 ± 0.15	0.82 ± 0.11	0.047
AA/LA	0.031 ± 0.0091*^#^	0.032 ± 0.0068*^#^	0.040 ± 0.0080	0.039 ± 0.0059	0.046
n-3 PUFAs	1.98 ± 0.18*^#^	1.95 ± 0.17*^#^	1.63 ± 0.13	1.69 ± 0.17	0.021
C18:3n-3 (ALA)	0.23 ± 0.042	0.25 ± 0.069	0.23 ± 0.036	0.23 ± 0.052	0.748
C20:5n-3 (EPA)	1.31 ± 0.20*^#^	1.31 ± 0.18*^#^	1.13 ± 0.14	1.12 ± 0.16	0.045
C22:6n-3 (DHA)	0.44 ± 0.031*^#^	0.39 ± 0.021*^#^	0.27 ± 0.041	0.34 ± 0.041	0.004
EPA/ALA	5.70 ± 1.09	5.24 ± 0.81	4.91 ± 0.74	4.87 ± 1.51	0.107
DHA/ALA	1.91 ± 0.19*^#^	1.56 ± 0.14*^#^	1.17 ± 0.16	1.27 ± 0.10	0.007
DHA/EPA	0.34 ± 0.051*^#^	0.30 ± 0.045*^#^	0.24 ± 0.081	0.25 ± 0.091	0.014
n-6/n-3 ∑PUFAs	11.14 ± 1.71*^#^	11.45 ± 2.01*^#^	12.38 ± 1.31	12.25 ± 2.01	0.024

*PUFAs* Polyunsaturated fatty acids, *LA* Linoleic acid, *ALA* α-Linolenic acid, *AA* Arachidonic acid, *EPA* Eicosapentaenoic acid, *DHA* Docosahexaenoic acid

*Compared with the control group, *P* < 0.05

^#^Compared with the S3 group, *P* < 0.05

^&^Data was analyzed by ANOVA (q test) among all these four groups. S1 group: exogenous DHA-rich n-3 PUFAs supplementation at the early pregnancy (0–12 week), S2 group: exogenous DHA-rich n-3 PUFAs supplementation at the middle pregnancy (13–27 week), S3 group: exogenous DHA-rich n-3 PUFAs supplementation at the late pregnancy (> 27 week), Control group: non-exogenous DHA-rich n-3 PUFAs supplementation during the whole pregnancy

### Effects of the 26 SNPs in the fatty acid desaturases and elongases on the profiles of PUFAs in the colostrum

The associations between the genotypes of 26 SNPs in the *Fads1, Fads2, Fads3, Elvol2* and *Elvol5,* and the profiles of n-6 PUFAs (LA and AA), n-3 PUFAs (ALA, EPA and DHA) and relative ratios (AA/LA, EPA/ALA, DHA/ALA, DHA/EPA and n-6/n-3ΣPUFAs) in the colostrum were analyzed using the SNPs stats software by adjusting the maternal age, gestational week, productive mode, infant sex and BMI at birth.

On the profiles of LA, AA and AA/LA in the colostrum, the carriers with effective T allele of *Fads3/*rs76996928 (b = − 1.638, *P* = 0.014) had lower LA than those with the C allele (TT < CT < CC). The subjects carrying the effective G allele of *Fads1/*rs174553 (b = − 0.050, *P* = 0.048) had lower AA than those with the homozygous A allele (GG < AG < AA), while the participants with the effective T allele in the *Fads1/*rs174550 ( b = 0.054, *P* = 0.038) and *Fads2/*rs174609 (b = 0.047, *P* = 0.013) had higher AA than those who carried the non-effective C allele (TT > CT > CC). Meanwhile, the subjects with effective T allele of *Fads1*/rs174550 (b = 0.003, *P* = 0.047) and *Fads3/*rs76996928 (b = 0.004, *P* = 0.022) had higher AA/LA than the subjects with C allele (TT > CT > CC) (Fig. 2B, Additional file 1: Tables S2, S3). As shown in the Fig. 2C, Additional file 1: Tables S4, S5), the subjects carrying the effective alleles of *Fads1/*rs174448 (A, b =  − 0.019, *P* = 0.046, AA < AG < GG), *Fads1/*rs174537 (G, b =  − 0.017, *P* = 0.031, GG < GT < TT), *Fads1/*rs174550 (T, b = − 0.017, *P* = 0.032, TT < CT < CC)*, Fads2/*rs174598 (A, b = − 0.018, *P* = 0.023, AA < AT < TT), *Fads2/*rs3168072 (A, b = − 0.025, *P* = 0.011, AA < AT < TT)*, Fads3*/rs174455 (C, b = − 0.020, *P* = 0.012, CC < CT < TT) and *Fads3*/rs174464 (G, b = − 0.019, *P* = 0.019, GG < AG < AA) had lower ALA, while the subjects with G allele in the *Fads1/*rs174553 (b = 0.018, *P* = 0.026) had higher ALA (GG > AG > AA) than those within the non-effective A allele. Meanwhile, the negative associations were shown between effective-alleles of *Fads1/*rs174550 (T, b = − 0.091, *P* = 0.045, TT < CT < CC) and *Fads2/*rs174598 (A, b = − 0.103, *P* = 0.019, AA < AT < TT) and the percentages of EPA in the colostrum, while the positive associations were observed between the G effective allele of *Fads1/*rs174553(b = 0.088, *P* = 0.047, GG > AG > AA) and higher EPA. Moreover, the carriers with effective alleles of *Fads3/*rs174455 (C, b = 0.012, *P* = 0.043, CC > CT > TT) and *Fads3/*rs174464 (G, b = 0.011, *P* = 0.048, GG > AG > AA) had higher DHA than those with the non-effective alleles. Furthermore, the negative associations were also demonstrated between effective A allele of *Fads1/*rs174448 (b = − 0.090, *P* = 0.021, AA < AG < GG) and *Fads2/*rs3168072 (b = − 0.081, *P* = 0.049, AA < AT < TT) with the ratio of DHA/EPA. However, no significant correlations were shown between the genotypes of 26 SNPs and the proportions of EPA/ALA, DHA/ALA and n-6/n-3 ΣPUFAs.

The effects of genetic risk scores (GRS) of the above 26 SNPs on the profiles of n-6 and n-3 PUFAs were shown in Table 4, there were significant correlations between GRS and the percents of LA (b = − 1.277, *P* = 0.018), AA (b = 0.115, *P* < 0.001), AA/LA (b = − 0.003, *P* < 0.001), ALA (b = − 0.023, *P* < 0.001), EPA (b = − 0.104, *P* < 0.001), DHA (b = 0.013, *P* = 0.015) and DHA/EPA (b = 0.089, *P* < 0.001) in the colostrum.

**Table 4:** Effects of genetic risk scores of the 26 SNPs in the fatty acid desaturases and elongases on the profiles of PUFAs in the colostrum

Indicators	b	SE	*P*
*n-6 PUFAs*			
LA	− 1.277	0.033	0.018
AA	0.115	0.017	< 0.001
AA/LA	− 0.003	0.004	< 0.001
*n-3 PUFAs*			
ALA	− 0.023	− 0.017	< 0.001
EPA	− 0.104	0.035	< 0.001
DHA	0.013	0.010	0.015
EPA/ALA	− 0.314	0.087	0.217
DHA/ALA	0.174	0.024	0.107
DHA/EPA	0.089	0.015	< 0.001
n-6/n-3 ΣPUFAs	0.136	0.038	0.243

*SNPs* Single nucleotide polymorphisms, *PUFAs* Polyunsaturated fatty acids, *LA* Linoleic acid, *AA* Arachidonic acid, *ALA* α-Linolenic acid, *EPA* Eicosapentaenoic acid, *DHA* Docosahexaenoic acid

### Interactions between maternal DHA-rich n-3 PUFAs supplementation and the genotypes of 26 SNPs on the profiles of PUFAs in the colostrum

The interactions between maternal DHA-rich n-3 PUFAs supplementation and the genotypes of 26 SNPs on the profiles of PUFAs were analyzed by adjusting the maternal age, gestational week, productive mode, infant sex and BMI at birth. As shown in Table 5, there were significant interactions between maternal exogenous DHA-rich n-3 PUFAs supplementation and the genotypes of significantly different SNPs on the percents of LA (*Fads1*/rs174448, *Fads1*/rs174553, *Fads2*/rs174598, *Fads3*/rs174464 and *Fads3*/ rs76996928), AA (*Fads2*/rs174609), ALA (*Elovl5*/rs209512) EPA (*Fads1*/rs174448 and *Fads2*/rs174598), DHA (*Fads1*/ rs174448, *Fads2*/rs174598 and *Fads2*/rs174602), EPA/ALA (*Elovl5*/rs209512), DHA/ALA (*Fads1*/rs174448 and *Fads2*/rs174619), DHA/EPA (*Fads1*/rs174448), and n-6/n-3ΣPUFAs (*Fads1*/rs174553, *Fads2*/rs174598, *Fads3*/rs174464 and *Fads3*/rs76996928).

**Table 5:** Interactions between maternal DHA-rich n-3 PUFAs supplementation and the genotypes of SNPs on the profiles of PUFAs in the colostrum (*P*_interaction_)

Gene	SNPs	n-6 PUFAs			n-3 PUFAs						n-6/n-3 ΣPUFAs
		LA	AA	AA/LA	ALA	EPA	DHA	EPA/ALA	DHA/ALA	DHA/EPA	
*Fads1*	rs174448	0.001	0.322	0.434	0.067	0.039	0.034	0.549	0.004	0.010	0.156
	rs174537	0.821	0.749	0.743	0.254	0.712	0.166	0.510	0.384	0.377	0.617
	rs174550	0.622	0.495	0.545	0.967	0.268	0.240	0.650	0.928	0.800	0.222
	rs174553	0.042	0.825	0.314	0.766	0.140	0.659	0.519	0.701	0.841	0.006
*Fads2*	rs174598	0.007	0.498	0.523	0.906	0.044	0.002	0.124	0.110	0.083	0.002
	rs174602	0.080	0.844	0.422	0.749	0.117	**0.030**	0.612	0.991	0.177	0.520
	rs174609	0.467	0.014	0.860	0.278	0.592	0.834	0.513	0.725	0.854	0.634
	rs174619	0.736	0.736	0.893	0.196	0.516	0.129	0.102	0.004	0.450	0.840
	rs498793	0.202	0.574	0.598	0.512	0.821	0.975	0.551	0.828	0.186	0.234
	rs3168072	0.435	0.069	0.988	0.318	0.846	0.298	0.994	0.223	0.492	0.461
*Fads3*	rs174455	0.074	0.864	0.817	0.824	0.217	0.399	0.748	0.989	0.162	0.129
	rs174464	0.005	0.907	0.757	0.811	0.878	0.746	0.872	0.423	0.668	0.003
	rs76996928	< 0.001	0.730	0.275	0.686	0.342	0.208	0.946	0.169	0.551	0.001
*Elovl2*	rs1323739	0.473	0.874	0.631	0.986	0.250	0.764	0.952	0.932	0.978	0.212
	rs2295602	0.976	0.491	0.819	0.185	0.206	0.687	0.357	0.356	0.505	0.510
	rs2180725	0.510	0.517	0.962	0.597	0.222	0.678	0.729	0.798	0.795	0.206
	rs3798710	0.427	0.709	0.654	0.341	0.287	0.510	0.583	0.484	0.789	0.142
	rs78793420	0.540	0.701	0.689	0.709	0.390	0.882	0.481	0.890	0.657	0.477
*Elovl5*	rs209512	0.264	0.931	0.213	0.021	0.732	0.106	0.047	0.268	0.712	0.941
	rs2281274	0.757	0.555	0.479	0.771	0.991	0.553	0.644	0.443	0.323	0.826
	rs2294852	0.639	0.441	0.992	0.726	0.314	0.968	0.791	0.735	0.445	0.346
	rs2397142	0.722	0.403	0.871	0.706	0.751	0.660	0.357	0.504	0.955	0.454
	rs6909592	0.949	0.524	0.650	0.711	0.306	0.445	0.500	0.412	0.535	0.449
	rs9349665	0.482	0.933	0.560	0.906	0.222	0.787	0.304	0.595	0.867	0.238
	rs9395858	0.252	0.801	0.795	0.844	0.279	0.889	0.227	0.794	0.698	0.177
	rs12207094	0.071	0.314	0.531	0.057	0.746	0.149	0.105	0.383	0.757	0.731

*SNPs* Single nucleotide polymorphisms, *Fads* Fatty acid desaturases, *Elovl* Elongase of long chain fatty acid, *PUFAs* Polyunsaturated fatty acids, *LA* Linoleic acid, *AA* Arachidonic acid, *ALA* α-Linolenic acid, *EPA* Eicosapentaenoic acid, *DHA* Docosahexaenoic acid

**Fig. 3:**
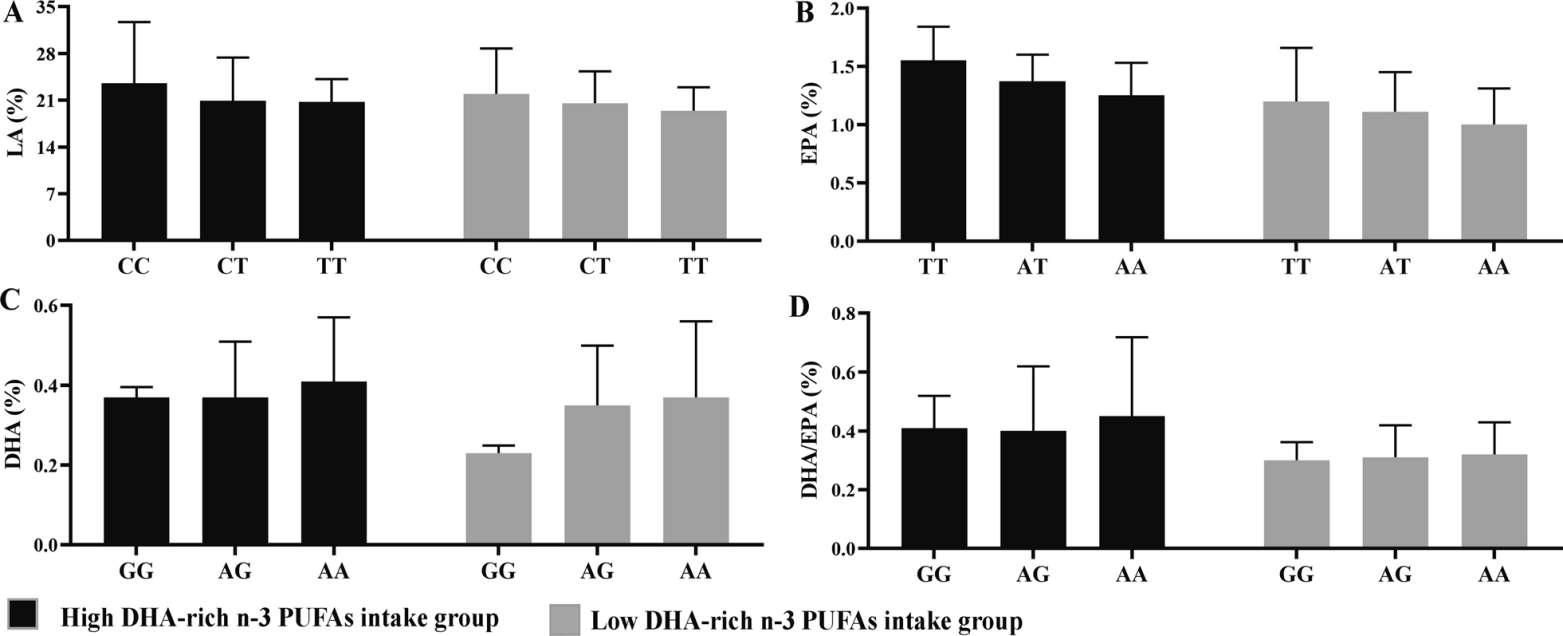
Interactions of maternal DHA-rich n-3PUFAs intake with the genotypes of significant SNPs in the fatty acid desaturases on the profiles of PUFAs in the colostrum.** A** presented the interactions of maternal DHA-rich n-3 PUFAs intake with *Fads*3/rs76996928 (T/C) on the content of LA.** B** presented the interactions of maternal DHA-rich n-3 PUFAs intake with *Fads2*/rs174598 (A/T) on the content of EPA.** C** and** D** respectively presented the interactions of maternal DHA-rich n-3 PUFAs intake with *Fads1*/rs174448 (A/G) on the ontents of DHA and DHA/EPA. Note: High DHA-rich n-3 PUFAs intake group was included maternal exogenous DHA-rich n-3 PUFAs supplementation at the early (S1) and middle (S2) pregnancy, Low DHA-rich n-3 PUFAs intake group was included maternal exogenous DHA-rich n-3 PUFAs supplementation at the late pregnancy (S3) and non-exogenous DHA-rich n-3 PUFAs supplementation during the whole pregnancy. *PUFAs* Polyunsaturated fatty acids, *SNPs* Single nucleotide polymorphisms, *Fads* Fatty acid desaturases, *LA* Linoleic acid, *EPA* Eicosapentaenoic acid, *DHA* Docosahexaenoic acid

To verify the above significant interactions, the subjects were divide into two groups, as high and low DHA-rich n-3 PUFAs intake groups. As shown in Table 6 and Fig. 3, there were significantly lower DHA (b = 0.040, *P* = 0.037, AA < AG < GG) and DHA/EPA (b = − 0.145, *P* = 0.015, AA < AG < GG) among the participants with the effective A allele in the *Fads1*/rs174448 under high DHA-rich n-3 PUFAs intake. Moreover, the carriers with the effective A allele in *Fads2*/rs174598 could decrease the percent of EPA than those with T allele under high DHA-rich n-3 PUFAs intake (b = − 0.139, *P* = 0.016, AA < AT < TT). However, in the low DHA-rich n-3 PUFAs intake group, compared with the subjects with non-effective allele of *Fads3*/rs76996928 C allele, T allele could decrease the contents of LA in the colostrum (b = − 1.759, *P* = 0.024, TT < CT < CC).

**Table 6:** Verification of the significant interactions between maternal DHA-rich n-3 PUFAs supplementation and genotype of 26 SNPs on the profiles of PUFAs

Gene	SNPs	High DHA-rich n-3 PUFAs intake group (n = 369)*			Low DHA-rich n-3 PUFAs intake group (n = 681)^#^		
		b (SE)	*t*	*P*	b (SE)	*t*	*P*
n-6 PUFAs							
LA *Fads1*	rs174448	1.807 (1.484)	1.218	0.229	1.045 (1.029)	1.016	0.313
*Fads1*	rs174553	0.626 (1.365)	0.459	0.649	0.237 (0.857)	0.277	0.782
*Fads2*	rs174598	− 0.522 (1.324)	− 0.394	0.695	− 0.076 (0.807)	− 0.094	0.926
*Fads3*	rs174464	1.488 (1.270)	1.171	0.247	− 0.255 (0.897)	− 0.285	0.777
*Fads3*	rs76996928	− 1.251 (1.228)	− 1.019	0.313	− 1.759 (0.763)	− 2.305	0.024
AA *Fads2*	rs174609	0.009 (0.045)	0.209	0.835	0.070 (0.045)	1.557	0.123
n-3 PUFAs							
ALA *Elovl5*	rs209512	− 0.019 (0.015)	− 1.237	0.222	− 0.013(0.010)	− 1.363	0.178
EPA *Fads1*	rs174448	0.052 (0.075)	0.695	0.491	0.111 (0.069)	1.611	0.111
*Fads2*	rs174598	− 0.139 (0.055)	− 2.507	0.016	− 0.104 (0.047)	− 1.197	0.072
DHA *Fads1*	rs174448	− 0.040 (0.041)	− 2.171	0.037	0.020 (0.037)	0.545	0.588
*Fads2*	rs174598	− 0.033 (0.033)	− 0.989	0.328	0.022 (0.030)	0.737	0.464
*Fads2*	rs174602	− 0.012 (0.036)	− 0.324	0.747	− 0.058 (0.032)	− 1.820	0.075
EPA/ALA *Elovl5*	rs209512	0.542 (0.426)	1.270	0.210	0.232 (0.282)	0.823	0.414
DHA/ALA *Fads1*	rs174448	0.052 (0.198)	0.263	0.793	0.038 (0.232)	0.165	0.869
*Fads2*	rs174619	0.130 (0.194)	0.672	0.505	0.359 (0.203)	1.767	0.082
DHA/EPA *Fads1*	rs174448	− 0.145 (0.059)	− 2.512	0.015	− 0.026 (0.050)	− 0.513	0.609
n-6/n-3Σ PUFAs							
*Fads1*	rs174553	− 0.264 (1.200)	− 0.220	0.827	0.070 (1.054)	0.067	0.947
*Fads2*	rs174598	0.537 (1.161)	0.463	0.645	0.241 (0.993)	0.243	0.809
*Fads3*	rs174464	0.821 (1.130)	0.726	0.471	0.155 (1.102)	0.141	0.888
*Fads3*	rs76996928	− 0.903 (1.077)	− 0.838	0.406	− 1.289 (0.942)	− 1.368	0.175

*Fads* Fatty acid desaturases, *Elovl* Elongase of long chain fatty acid, *PUFAs* Polyunsaturated fatty acids, *SNPs* Single nucleotide polymorphisms, *LA* Linoleic acid, *AA* Arachidonic acid, *ALA* α-Linolenic acid, *EPA* Eicosapentaenoic acid, *DHA* Docosahexaenoic acid

*High DHA-rich n-3 PUFAs intake group was included maternal exogenous DHA-rich n-3 PUFAs supplementation at the early (S1) and middle (S2) pregnancy

^#^Low DHA-rich n-3 PUFAs intake group was included maternal exogenous DHA-rich n-3 PUFAs supplementation at the late pregnancy (S3) and non-exogenous DHA-rich n-3 PUFAs supplementation during the whole pregnancy

## Discussion

Mounting epidemiological investigations have shown that DHA can rapidly accumulate in the maternal neural cortex, retinal membrane synapses and adipose tissues from the early to late pregnancy [19], which mainly comes from the dietary and exogenous DHA-rich n-3 PUFAs supplementation by the placenta and umbilical cord to their fetus. Our previous study also had proved that there were positive correlations between the profiles of PUFAs in the colostrum and umbilical cord blood to some extent [18], so it is feasible and meaningful that the colostrum was chosen as the target biological samples. Meanwhile, the remarkable associations between maternal DHA intake during the pregnancy and circulating DHA showed that the dietary and exogenous DHA-rich n-3 PUFAs supplementation might be a major source of DHA [19], which was consistent with our results that maternal exogenous DHA-rich n-3 PUFAs supplementation was important because the dietary DHA intake was less than its recommended intake. Thus, as expected, pregnant women who were supplemented with more than 300 mg exogenous DHA supplementation per day from the early and middle pregnancy could reduce the infant BMI at birth, and impact the profiles of PUFAs (higher n-3 PUFAs: EPA, DHA, DHA/ALA, DHA/EPA, lower n-6 PUFAs: AA, AA/LA, and n-6/n-3ΣPUFAs) in the colostrum using this birth cohort with the 1050 mother-infant pairs, which was in agreement with the previous epidemiological studies [20, 21].

According to the Chinese dietary structure and limited exogenous DHA-rich n-3 PUFAs supplementation [7, 8], the endogenous synthesis has become an important pathway to increase the content of DHA in the colostrum, which are mainly regulated by the FADS and ELOVL. To date, many previous findings had supported that more than 20% variability of both n-6 and n-3 PUFAs were associated with the genotypes of SNPs in the *Fads1*, *Fads2*, *Fads3*, *Elovl2* and *Elovl5* [22]. However, the previous studies regarding the homozygous influences of various SNPs on the profiles of n-6 and n-3 PUFAs had produced the conflicting results, in which some had proved there were significant associations between the genotypes of SNPs and the profiles of PUFAs, especially EPA and DHA, in the colostrum, plasma and other tissues [23–28], the others did not find the above related correlations [29, 30]. Exactly, Xie and Innis observed there were significantly lower proportion of ALA, but not DHA among the subjects with the minor allele homozygotes of *Fads1/*rs174553 [9]. Wolters et al. also proved that there were strong associations between the effective alleles of SNPs in the *Fads1* (rs174537, rs174545, rs174546, rs174547, rs174553, rs174556, rs174561, rs174568 and rs99780) and the concentrations of AA, EPA and DHA in the plasma, which was positively consistent with the lower transcriptions of FADS and ELOVL to explain the increasing proportions of PUFAs [31–33]. Simultaneously, the pregnant women who were homozygous with the minor alleles of SNPs (rs174570, rs174574, rs174575, rs174576, rs174598, rs174579, rs174602, rs498793, rs2727271 and rs3834458) in the *Fads2* had lower concentrations of ALA, EPA and DHA in the clinical trial using the GWAS [29]. Moreover, the genotypes of SNPs (rs174570, rs174576, rs174679, rs174611, rs174593, rs174626, rs207214, rs2845573 and rs2851682) in the *Fads2* could significantly impact the contents of n-6 and n-3 PUFAs among the white people. Meanwhile, among the East Asian women, those correlations were shown with the genotypes of SNPs (rs174602, rs174626, rs207214, rs2845573 and rs2851682) in the *Fads2* [17, 34, 35]. Furthermore, the genotypes of SNPs (rs174550, rs174464, rs7115739 and rs1000778) in the *Fads3* were associated with the plasma concentrations of AA, EPA and DHA [36, 37], so it is difficult to explain these conflicting results in the literature due to different DHA supplementation, different populations and so on. Therefore, following the strict inclusion criteria, the subjects in our study were divided into different groups according to maternal exogenous DHA-rich n-3 PUFAs supplementation to improve the existing inconsistencies, in which our data suggested that the profiles of n-6 (LA, AA and AA/LA) and n-3 PUFAs (ALA, EPA, DHA, EPA/ALA, DHA/ALA and DHA/EPA) in the colostrum could be regulated by the genotypes of SNPs in the FADS and ELOVL, such as rs174448 (A/G), rs174537 (G/T), rs174550 (T/C) and rs174553 (G/A) in the *Fads1*, rs174598 (A/T) and rs3168072 (A/T) in the *Fads2,* and rs174455 (C/T), rs174464(G/A) and rs76996928 (T/C) in the *Fads3* (*P* < 0.05), which were in agreement with the previous studies [23–28]. However, the genotypes of SNPs in the *Elovl2* and *Elovl5* were not associated with the profiles of PUFAs in the colostrum using our birth cohort (*P* > 0.05) [27, 28, 37].

To our knowledge, the different profiles of PUFAs in the colostrum might contribute to the better understanding interactions between the significant genotypes of SNPs in the *Fads1, Fads2*, *Fads3*, *Elovl2* and *Elovl5*, and maternal exogenous DHA supplementation. Interestingly, under the high exogenous DHA intake, no differences were found in the percent of AA, with the significant differences in the percentages of LA, EPA and DHA among the pregnant women, who carried the effective minor alleles in the FADS and ELOVL. As Xie and Innis discussed the maternal dietary DHA intake, in which the possible explanation for our differences was that our population had the lower usual DHA status than the others [9]. However, the lower DHA status was not observed among the subjects with the minor allele homozygotes (rs174553) of the *Fads1* at baseline in the control group at birth after exogenous DHA supplementation. And the carriers with minor allele in the *Fads2/*rs174602 were at the greater risk of DHA deficiency [27]. Moreover, Dumitrescu L et al. also found that the concentrations of DHA were higher among the population with *Fads1* rs174448 and rs174550 than the other genotypes of SNPs by the exogenous fish oil intervention [37], which was consistent with our study that there were meaningful interactions between maternal DHA-rich n-3 PUFAs supplementation and the related genotypes of SNPs (rs76996928 for LA, rs174598 for EPA, and rs174448 for DHA and DHA/EPA) on the profiles of PUFAs. Totally, the major novelty of this study was that the pregnant women with the genotypes of SNPs (*Fads3/*rs174455-T, *Fads3*/rs174464-A and *Fads1/*rs174448 G alleles) should pay more attention on the exogenous DHA supplementation from the early and middle pregnancy for their blocked endogenous synthesis. If so, further interactions were warranted on whether these findings might have any functional consequences for the development and health of the children.

There were still some limitations in this study: Firstly, the consumption of maternal dietary and exogenous DHA-rich n-3 PUFAs supplementation was from the retrospective FFQ, so the calculation of DHA intake was not accurate enough for the different brands and doses of exogenous DHA-rich n-3 PUFAs supplementation, so more clinical studies should be chosen to strictly control the precise exogenous DHA supplementation using the birth cohorts. Moreover, the above 26 SNPs were chosen as the candidate genotypesby the MALDI-TOF–MS from the references rather than using an un-biased whole-genome genotyping or targeted sequencing, in which the true causal variants that had functional impacts could have been missed in the analysis, so more whole-genome genotyping studies should be used in the future. Furthermore, we anticipated that the increases in the dietary variability would tend to blur the potential effects of SNPs with small population, more birth cohorts with much larger samples will need to be verified the conclusion of our study. Finally, the activity and transcriptions of FADS and ELOVL should be measured to more clearly explain the biological function of the related SNPs on the profiles of PUFAs in the colostrum.

## Conclusions

In summary, results from this birth cohort proved that the pregnant women with the genotypes of the SNPs such as *Fads1/*rs174448 G, *Fads3/*rs174455 T and *Fads3/*rs174464 A alleles should pay more attention on their exogenous DHA-rich n-3 PUFAs supplementation from the early and middle pregnancy for their blocked endogenous synthesis to improve the fitness of their infants. Therefore, the implementation of this project can instruct the personalized exogenous DHA supplementation among the pregnant women, which not only ensure the reasonable utilization of exogenous DHA supplementation, but also meet the needs of fetus to truly reflect the fairness and justice of public health.

## Supplementary Information

**Additional file 1. Table S1.** Basic information of the genotypes of SNPs in the fatty acid desaturases and elongases. **Table S2.** Effects of the 26 SNPs in the fatty acid desaturases and elongases on the percents of LA and AA in the colostrum. **Table S3.** Effects of the 26 SNPs in the fatty acid desaturases and elongases on the AA/LA in the colostrum. **Table S4.** Effects of the 26 SNPs in the fatty acid desaturases and elongases on the profiles of n-3 PUFAs in the colostrum. **Table S5.** Effects of the 26 SNPs in the fatty acid desaturases and elongases on the related ratios of n-6 and n-3 PUFAs in the colostrum. **Table S6.** Distributions of the 26 SNPs in the fatty acid desaturases and elongases of the subjects in different groups.

## Data Availability

The data and materials that support the findings of this study are available from the corresponding author upon the reasonable requests.

## Abbreviations

PUFAs: Polyunsaturated fatty acids
DHA: Docosahexaenoic acid
LA: Linoleic acid
ALA: α-Linolenic acid
AA: Arachidonic acid
EPA: Eicosapentaenoic acid
SNPs: Single nucleotide polymorphisms
*Fads*: Fatty acid desaturases
*Elovl*: Elongase of very long chain fatty acid
FFQ: Food frequency questionnaire
GC: Gas chromatography
LD: Linkage disequilibrium
MALDI-TOF-MS: Matrix-assisted laser desorption ionization time of flight mass spectrometry
MAF: Minor allele frequencies
ANOVA: Analysis of variance
