# Supplementary material for: Maternal DHA-rich n-3 PUFAs supplementation interacts with FADS genotypes to influence the profiles of PUFAs in the colostrum among Chinese Han population: a birth cohort study
Source: Nutr Metab (Lond). 2022 Jul 23;19:48. doi: 10.1186/s12986-022-00683-3 (PMC9308251; doi:10.1186/s12986-022-00683-3)
Supplement: Supplementary file 1 — Additional file 1. Table S1. Basic information of the genotypes of SNPs in the fatty acid desaturases and elongases. Table S2. Effects of the 26 SNPs in the fatty acid desaturases and elongases on the percents of LA and AA in the colostrum. Table S3. Effects of the 26 SNPs in the fatty acid desaturases and elongases on the AA/LA in the colostrum. Table S4. Effects of the 26 SNPs in the fatty acid desaturases and elongases on the profiles of n-3 PUFAs in the colostrum. Table S5. Effects of the 26 SNPs in the fatty acid desaturases and elongases on the related ratios of n-6 and n-3 PUFAs in the colostrum. Table S6. Distributions of the 26 SNPs in the fatty acid desaturases and elongases of the subjects in different groups. [file 12986_2022_683_MOESM1_ESM.doc]

**Supplementary Table 1 Basic information of the genotypes of SNPs**

**in the fatty acid desaturase and elongases**

| Gene | SNPs | Position^&^ | MAF* | Forward primer | Reverse primer | Extension primer |
| --- | --- | --- | --- | --- | --- | --- |
| *Fads1* | rs174448 | 11:61872101 | G: 0.350 | ACGTTGGATGGTCTGGGAGATTATGTGAGC | ACGTTGGATGATGACAGACAGGGTGTTTGG | CTTCCCTCACCCTGA |
|  | rs174537 | 11:61785208 | T: 0.337 | ACGTTGGATGGGGTGCTTCTGAGGGAGG | ACGTTGGATGCCATGTCTGCTGTGTGTCTA | tagcgGGGCTCTCCCTCTGTCTTGGAC |
|  | rs174550 | 11: 61804006 | C: 0.341 | ACGTTGGATGCCCTTCAAAAGTACCAAGGC | ACGTTGGATGCATTTGGCGGCATGCTGAAG | tctgtGCTAGGAAGAGCTAGAGGCAA |
|  | rs174553 | 11: 61807686 | A: 0.297 | ACGTTGGATGTAAGCCGAGCTGCTGAAGAG | ACGTTGGATGAGCAGAACTTTGCTGTCTTG | AGCCAGTCTAGAACCCCTG |
| *Fads2* | rs174598 | 11:61853722 | T: 0.344 | ACGTTGGATGACAGGCTTAAGCCACCCATC | ACGTTGGATGACACAGAGAGAAACAGGCGG | ggaggCACATGCCGCCCTCTC |
|  | rs174602 | 11: 61856942 | T: 0.199 | ACGTTGGATGAGCAGATAGAAGGGATGGTG | ACGTTGGATGAGAGGAGAGATCATGAGCAC | gacatCCTCCCATGTTCCCCAACCC |
|  | rs174609 | 11:61860339 | C: 0.441 | ACGTTGGATGAGGAAAGAGGTGACAAACCC | ACGTTGGATGAACAGGGAACCTTGCCAGAG | GGGAAGCCTCCTTCA |
|  | rs174619 | 11:61862194 | A: 0.438 | ACGTTGGATGGGCTGAGTAAAGACAGGTTC | ACGTTGGATGCCAACTCTCCCCTGCTAAAG | CCCTGCTAAAGCCCCCA |
|  | rs498793 | 11: 61857233 | T: 0.313 | ACGTTGGATGCTAAACTTGTTAGAAGCGGG | ACGTTGGATGCTCTGTGTCCCCATCAGGC | gtgttCAGACCCCTCAGCCTG |
|  | rs3168072 | 11: 61864038 | T: 0.256 | ACGTTGGATGCACTTGGTCATAGTGGTTAC | ACGTTGGATGTGTGCAGGCTCCATAACTAC | aggtGTTACCACCTCCCTCC |
| *Fads3* | rs174455 | 11: 61888710 | T: 0.279 | ACGTTGGATGATAAGGTCACCCAGGAAAGG | ACGTTGGATGAAATGCTGCTGGCCCCTAAG | ctGCTGTCCCACCATAC |
|  | rs174464 | 11: 61890454 | A: 0.261 | ACGTTGGATGATTTGGAGGGTCAAGGACAG | ACGTTGGATGGAGTCCAGTGGTGGAGCAG | GCCCCAACCCCTATT |
|  | rs76996928 | 11: 61883454 | C: 0.403 | ACGTTGGATGTGATGGTGAGTCCCTCTTTC | ACGTTGGATGTGGGCTGGTGGGTGTGTCT | ctcccTTGAAACTCCCCAGACACACA |
| *Elovl2* | rs1323739 | 6: 11004328 | C: 0.401 | ACGTTGGATGCTCAAGATCACCAGAAAGGC | ACGTTGGATGATACAAGCTATAAGCGGTGG | ggggAAGCTATAAGCGGTGGATAATG |
|  | rs2295602 | 6:11005609 | T: 0.317 | ACGTTGGATGAGGATCCTGAGGAATGATGC | ACGTTGGATGTGGAGTTGTTCCCTGTTGTG | GGAATGATGCTCCTGTT |
|  | rs2180725 | 6: 11025187 | C: 0.189 | ACGTTGGATGCACCATTCCTTGAAGAGAAA | ACGTTGGATGATGGAGAGTGGGAACCCGAG | gggcGGGCTTGAATCTGCT |
|  | rs3798710 | 6: 11002550 | C: 0.251 | ACGTTGGATGCACAATCAAAGAGAGGAGAC | ACGTTGGATGACCCACTCTTTCTCTTAAAC | GCAATCAATGGTAGTGATTTT |
|  | rs78793420 | 6: 11039168 | C: 0.153 | ACGTTGGATGATGTAAAGATGCTGAGGCCC | ACGTTGGATGGACACTGTTTACTCACTTGC | cttgaTAAATGGTGTATTATCTCTGTC |
| *Elovl5* | rs209512 | 6: 53338779 | G: 0.202 | ACGTTGGATGCATTTTCTGCTACAGGCAAC | ACGTTGGATGCAAGTAATCACATCAGTTAAG | ACATCAGTTAAGAGACTGC |
|  | rs2281274 | 6: 53278756 | C: 0.215 | ACGTTGGATGTCTTCCACCTGTTGAGGTTC | ACGTTGGATGAGTGTGGTAGGTGTGAGTAG | CGGAGCTCTGTCATGCT |
|  | rs2294852 | 6: 53292416 | G: 0.423 | ACGTTGGATGACATGCAGACTCAGAAAACG | ACGTTGGATGCATAGCAAAGGCTGCCTTTC | agggCTAATTAGTGCCAGTTTCACTAAC |
|  | rs2397142 | 6: 53335501 | G: 0.357 | ACGTTGGATGTGCTTAGTCTCTAGGCAACG | ACGTTGGATGCGAGTAAACAGGAAGGACAC | gggctTTTCCTCATCCCCTCCC |
|  | rs6909592 | 6: 53298292 | C: 0.408 | ACGTTGGATGGCATCCCAAACAGCCAAATG | ACGTTGGATGTCCCCAGTGAGCAGCTTGT | taaatGCTATGACTAATGGATTGAATAA |
|  | rs9349665 | 6: 53341701 | C: 0.220 | ACGTTGGATGTTGCAGAATATTCCGTTGCC | ACGTTGGATGGATTGGAAGAGTTAGTAGAC | CCTTGAAAAGAATAAATCATGCT |
|  | rs9395858 | 6: 53313689 | T: 0.219 | ACGTTGGATGCACCATGCCTGGCTAAAAAC | ACGTTGGATGGGTAAACTTTTATTATTTTTC | tcTTTTTCATTTTCAAAAACATCTG |
|  | rs12207094 | 6: 53339377 | T: 0.138 | ACGTTGGATGTGTTGCCAAACCTAGTGTCC | ACGTTGGATGACCACCTTCAGTTCTCTTAG | gggtgCTCTTAGGATCCTTAGACTC |

Note：*MAF: Minor allele frequency. **^&^**The position in the Chromosome. SNPs: Single nucleotide polymorphisms, *Fads*: fatty acid desaturases, *Elovl*: elongase of long chain fatty acid.

**Supplementary Table 2 Effects of the 26 SNPs in the fatty acid desaturases and elongases on the percents of LA and AA in the colostrum**

| Gene | SNPs | LA | | | |  | AA | | | |
| --- | --- | --- | --- | --- | --- | --- | --- | --- | --- | --- |
|  |  | b | SE | *t* | *P* |  | b | SE | *t* | *P* |
| *Fads1* | rs174448 | 1.304 | 0.850 | 1.534 | 0.127 |  | 0.023 | 0.031 | 0.759 | 0.449 |
|  | rs174537 | -0.333 | 0.698 | -0.477 | 0.634 |  | 0.045 | 0.025 | 1.805 | 0.073 |
|  | rs174550 | -0.473 | 0.724 | -0.653 | 0.515 |  | **0.054** | **0.026** | **2.095** | **0.038** |
|  | rs174553 | 0.404 | 0.736 | 0.549 | 0.584 |  | **-0.050** | **0.026** | **-2.012** | **0.048** |
| *Fads2* | rs174598 | -0.245 | 0.700 | -0.350 | 0.727 |  | 0.041 | 0.025 | 1.657 | 0.100 |
|  | rs174602 | -0.004 | 0.780 | -0.005 | 0.996 |  | -0.048 | 0.032 | -1.500 | 0.136 |
|  | rs174609 | 0.585 | 0.904 | 0.648 | 0.518 |  | **0.047** | **0.028** | **2.690** | **0.013** |
|  | rs174619 | 0.050 | 0.804 | 0.063 | 0.950 |  | 0.022 | 0.029 | 0.770 | 0.443 |
|  | rs498793 | 0.425 | 1.334 | 0.318 | 0.751 |  | 0.021 | 0.048 | 0.437 | 0.662 |
|  | rs3168072 | 0.985 | 0.906 | 1.087 | 0.279 |  | 0.011 | 0.033 | 0.349 | 0.728 |
| *Fads3* | rs174455 | -0.455 | 0.718 | -0.635 | 0.527 |  | 0.021 | 0.026 | 0.795 | 0.428 |
|  | rs174464 | 0.488 | 0.739 | 0.660 | 0.510 |  | 0.016 | 0.027 | 0.596 | 0.552 |
|  | rs76996928 | **-1.638** | **0.655** | **-2.502** | **0.014** |  | 0.045 | 0.024 | 1.875 | 0.063 |
| *Elovl2* | rs1323739 | -0.753 | 0.688 | -1.095 | 0.276 |  | 0.001 | 0.025 | 0.053 | 0.958 |
|  | rs2295602 | -1.420 | 1.104 | -1.286 | 0.201 |  | 0.019 | 0.040 | 0.476 | 0.635 |
|  | rs2180725 | 0.003 | 0.780 | 0.004 | 0.997 |  | 0.005 | 0.029 | 0.188 | 0.851 |
|  | rs3798710 | 0.729 | 0.655 | 1.113 | 0.268 |  | <0.001 | 0.024 | 0.018 | 0.985 |
|  | rs78793420 | -0.189 | 0.752 | -0.252 | 0.802 |  | -0.001 | 0.027 | -0.047 | 0.963 |
| *Elovl5* | rs209512 | 0.229 | 0.725 | 0.315 | 0.753 |  | -0.038 | 0.026 | -1.456 | 0.148 |
|  | rs2281274 | 0.837 | 0.782 | 1.070 | 0.287 |  | -0.014 | 0.028 | -0.480 | 0.632 |
|  | rs2294852 | -0.382 | 0.776 | -0.492 | 0.623 |  | -0.025 | 0.028 | -0.886 | 0.377 |
|  | rs2397142 | 0.108 | 0.774 | 0.139 | 0.890 |  | -0.040 | 0.028 | -1.449 | 0.150 |
|  | rs6909592 | -0.315 | 0.780 | -0.404 | 0.687 |  | -0.027 | 0.028 | -0.970 | 0.334 |
|  | rs9349665 | -0.123 | 0.785 | -0.156 | 0.876 |  | -0.037 | 0.028 | -1.313 | 0.192 |
|  | rs9395858 | -0.009 | 0.717 | -0.012 | 0.990 |  | -0.020 | 0.026 | -0.759 | 0.449 |
|  | rs12207094 | -0.488 | 1.074 | -0.455 | 0.650 |  | -0.021 | 0.039 | -0.554 | 0.581 |

Note: LA: linoleic acid, AA: arachidonic acid, SNPs: Single nucleotide polymorphisms, *Fads*: fatty acid desaturases, *Elovl*: elongase of long chain fatty acid.

**Supplementary Table 3 Effects of the 26 SNPs in the fatty acid desaturases and elongases on the AA/LA in the colostrum**

| Gene | SNPs | AA/LA | | | |
| --- | --- | --- | --- | --- | --- |
|  |  | b | SE | *t* | *P* |
| *Fads1* | rs174448 | -0.002 | 0.002 | -1.069 | 0.287 |
|  | rs174537 | 0.002 | 0.002 | 1.451 | 0.149 |
|  | **rs174550** | **0.003** | **0.002** | **2.021** | **0.047** |
|  | rs174553 | -0.003 | 0.002 | -1.537 | 0.127 |
| *Fads2* | rs174598 | 0.002 | 0.002 | 1.250 | 0.213 |
|  | rs174602 | -0.003 | 0.002 | -1.459 | 0.147 |
|  | rs174609 | <0.001 | 0.002 | 0.142 | 0.887 |
|  | rs174619 | <0.001 | 0.002 | -0.322 | 0.748 |
|  | rs498793 | 0.001 | 0.003 | 0.169 | 0.866 |
|  | rs3168072 | -0.002 | 0.002 | -0.824 | 0.411 |
| *Fads3* | rs174455 | 0.001 | 0.002 | 0.636 | 0.528 |
|  | rs174464 | <0.001 | 0.002 | 0.141 | 0.888 |
|  | **rs76996928** | **0.004** | **0.002** | **2.315** | **0.022** |
| *Elovl2* | rs1323739 | <0.001 | 0.002 | -0.237 | 0.813 |
|  | rs2295602 | 0.003 | 0.003 | 1.197 | 0.233 |
|  | rs2180725 | 0.002 | 0.002 | 0.891 | 0.374 |
|  | rs3798710 | <0.001 | 0.002 | -0.373 | 0.710 |
|  | rs78793420 | -0.002 | 0.002 | -0.927 | 0.356 |
| *Elovl5* | rs209512 | -0.002 | 0.002 | -1.050 | 0.296 |
|  | rs2281274 | -0.002 | 0.002 | -1.054 | 0.294 |
|  | rs2294852 | <0.001 | 0.002 | -0.457 | 0.648 |
|  | rs2397142 | -0.002 | 0.002 | -1.213 | 0.227 |
|  | rs6909592 | -0.001 | 0.002 | -0.598 | 0.551 |
|  | rs9349665 | -0.001 | 0.002 | -0.795 | 0.428 |
|  | rs9395858 | <0.001 | 0.002 | -0.564 | 0.574 |
|  | rs12207094 | 0.001 | 0.002 | 0.598 | 0.551 |

Note: LA: linoleic acid, AA: arachidonic acid, SNPs: Single nucleotide polymorphisms. *Fads*: fatty acid desaturases, *Elovl*: elongase of long chain fatty acid.

**Supplementary Table 4 Effects of the 26 SNPs in the fatty acid desaturases and elongases on the percentages of**

**n-3 PUFAs in the colostrum**

| Gene | SNPs | ALA | | | |  | EPA | | | |  | DHA | | | |
| --- | --- | --- | --- | --- | --- | --- | --- | --- | --- | --- | --- | --- | --- | --- | --- |
|  |  | b | SE | *t* | *P* |  | b | SE | *t* | *P* |  | b | SE | *t* | *P* |
| *Fads1* | rs174448 | **-0.019** | **0.009** | **-2.014** | **0.046** |  | 0.079 | 0.054 | 1.471 | 0.144 |  | -0.013 | 0.028 | -0.443 | 0.659 |
|  | rs174537 | **-0.017** | **0.008** | **-2.279** | **0.031** |  | -0.065 | 0.044 | -1.481 | 0.141 |  | 0.009 | 0.024 | 0.378 | 0.706 |
|  | rs174550 | **-0.017** | **0.008** | **-2.171** | **0.032** |  | **-0.091** | **0.045** | **-2.028** | **0.045** |  | <0.001 | 0.025 | -0.005 | 0.996 |
|  | rs174553 | **0.018** | **0.008** | **2.259** | **0.026** |  | **0.088** | **0.046** | **2.022** | **0.047** |  | 0.001 | 0.025 | 0.005 | 0.996 |
| *Fads2* | rs174598 | **-0.018** | **0.008** | **-2.313** | **0.023** |  | **-0.103** | **0.043** | **-2.380** | **0.019** |  | -0.011 | 0.024 | -0.445 | 0.657 |
|  | rs174602 | 0.010 | 0.009 | 1.202 | 0.232 |  | 0.032 | 0.049 | 0.648 | 0.518 |  | -0.030 | 0.026 | -1.134 | 0.259 |
|  | rs174609 | -0.027 | 0.010 | -1.815 | 0.056 |  | 0.033 | 0.057 | 0.584 | 0.560 |  | -0.008 | 0.030 | -0.262 | 0.794 |
|  | rs174619 | -0.026 | 0.009 | -0.734 | 0.463 |  | 0.023 | 0.051 | 0.462 | 0.645 |  | 0.005 | 0.028 | 0.186 | 0.853 |
|  | rs498793 | -0.007 | 0.015 | -0.466 | 0.642 |  | 0.011 | 0.084 | 0.137 | 0.892 |  | 0.019 | 0.046 | 0.419 | 0.676 |
|  | rs3168072 | **-0.025** | **0.010** | **-2.577** | **0.011** |  | 0.030 | 0.057 | 0.516 | 0.607 |  | -0.015 | 0.030 | -0.512 | 0.610 |
| *Fads3* | rs174455 | **-0.020** | **0.008** | **-2.545** | **0.012** |  | -0.011 | 0.045 | -0.254 | 0.800 |  | **0.012** | **0.024** | **2.073** | **0.043** |
|  | rs174464 | **-0.019** | **0.008** | **-2.374** | **0.019** |  | -0.003 | 0.047 | -0.057 | 0.954 |  | **0.011** | **0.024** | **2.006** | **0.048** |
|  | rs76996928 | -0.019 | 0.007 | -0.510 | 0.614 |  | -0.053 | 0.042 | -1.276 | 0.204 |  | 0.012 | 0.023 | 0.545 | 0.587 |
| *Elovl2* | rs1323739 | 0.004 | 0.008 | 0.497 | 0.620 |  | -0.052 | 0.043 | -1.193 | 0.235 |  | 0.008 | 0.025 | 0.339 | 0.735 |
|  | rs2295602 | -0.011 | 0.013 | -0.829 | 0.409 |  | -0.073 | 0.070 | -1.041 | 0.300 |  | 0.017 | 0.041 | 0.423 | 0.673 |
|  | rs2180725 | -0.003 | 0.009 | -0.276 | 0.783 |  | 0.036 | 0.049 | 0.730 | 0.467 |  | 0.017 | 0.027 | 0.624 | 0.534 |
|  | rs3798710 | <0.001 | 0.008 | -0.031 | 0.975 |  | 0.034 | 0.041 | 0.828 | 0.409 |  | -0.017 | 0.024 | -0.715 | 0.477 |
|  | rs78793420 | 0.009 | 0.009 | 1.028 | 0.306 |  | -0.025 | 0.047 | -0.526 | 0.600 |  | 0.007 | 0.026 | 0.279 | 0.781 |
| *Elovl5* | rs209512 | -0.014 | 0.009 | -1.678 | 0.096 |  | 0.033 | 0.046 | 0.724 | 0.470 |  | 0.019 | 0.026 | 0.727 | 0.469 |
|  | rs2281274 | <0.001 | 0.009 | -0.080 | 0.936 |  | -0.017 | 0.049 | -0.347 | 0.729 |  | 0.018 | 0.027 | 0.675 | 0.501 |
|  | rs2294852 | <0.001 | 0.009 | -0.028 | 0.978 |  | 0.015 | 0.049 | 0.301 | 0.764 |  | -0.006 | 0.026 | -0.228 | 0.820 |
|  | rs2397142 | 0.001 | 0.009 | 0.129 | 0.900 |  | -0.005 | 0.049 | -0.104 | 0.918 |  | -0.014 | 0.026 | -0.518 | 0.606 |
|  | rs6909592 | -0.002 | 0.009 | -0.258 | 0.797 |  | 0.007 | 0.049 | 0.138 | 0.890 |  | -0.004 | 0.026 | -0.145 | 0.885 |
|  | rs9349665 | 0.001 | 0.009 | 0.068 | 0.946 |  | -0.006 | 0.049` | -0.116 | 0.908 |  | -0.004 | 0.027 | -0.138 | 0.891 |
|  | rs9395858 | -0.006 | 0.009 | -0.706 | 0.482 |  | -0.011 | 0.045 | -0.238 | 0.812 |  | 0.013 | 0.025 | 0.516 | 0.607 |
|  | rs12207094 | 0.002 | 0.012 | 0.199 | 0.842 |  | 0.017 | 0.067 | 0.252 | 0.801 |  | 0.040 | 0.036 | 1.124 | 0.264 |

Note: ALA: α-linolenic acid, EPA: eicosapentaenoic acid, DHA: docosahexaenoic acid, SNPs: Single nucleotide polymorphisms. *Fads*: fatty acid desaturases, *Elovl*: elongase of long chain fatty acid.

**Supplementary Table 5 Effects of the 26 SNPs in the fatty acid desaturases and elongases on the related ratio**

**of n-6 and n-3 PUFAs in the colostrum**

| Gene | SNPs | EPA/ALA | | | |  | DHA/ALA | | | |  | DHA/EPA | | | |  | **n-6/n-3 ΣPUFAs** | | | |
| --- | --- | --- | --- | --- | --- | --- | --- | --- | --- | --- | --- | --- | --- | --- | --- | --- | --- | --- | --- | --- |
|  |  | b | SE | *t* | *P* |  | b | SE | *t* | *P* |  | b | SE | *t* | *P* |  | b | SE | *t* | *P* |
| *Fads1* | rs174448 | 0.377 | 0.316 | 1.192 | 0.236 |  | 0.006 | 0.164 | 0.039 | 0.969 |  | **-0.090** | **0.038** | **-2.353** | **0.021** |  | -0.188 | 0.977 | -0.193 | 0.848 |
|  | rs174537 | -0.194 | 0.263 | -0.739 | 0.461 |  | 0.150 | 0.135 | 1.106 | 0.271 |  | -0.015 | 0.034 | -0.435 | 0.665 |  | -0.108 | 0.795 | -0.136 | 0.892 |
|  | rs174550 | -0.354 | 0.264 | -1.340 | 0.183 |  | 0.126 | 0.137 | 0.919 | 0.360 |  | -0.018 | 0.034 | -0.538 | 0.592 |  | 0.054 | 0.825 | 0.065 | 0.948 |
|  | rs174553 | 0.325 | 0.269 | 1.209 | 0.229 |  | -0.103 | 0.139 | -0.742 | 0.459 |  | 0.018 | 0.034 | 0.538 | 0.592 |  | -0.079 | 0.838 | -0.094 | 0.925 |
| *Fads2* | rs174598 | -0.393 | 0.254 | -1.546 | 0.125 |  | 0.109 | 0.132 | 0.822 | 0.413 |  | -0.016 | 0.033 | -0.489 | 0.626 |  | 0.338 | 0.796 | 0.425 | 0.671 |
|  | rs174602 | 0.212 | 0.288 | 0.735 | 0.464 |  | -0.117 | 0.148 | -0.788 | 0.432 |  | -0.020 | 0.036 | -0.556 | 0.580 |  | 0.114 | 0.888 | 0.128 | 0.898 |
|  | rs174609 | 0.273 | 0.333 | 0.713 | 0.477 |  | 0.085 | 0.172 | 0.493 | 0.623 |  | -0.075 | 0.041 | -1.836 | 0.069 |  | 0.041 | 1.031 | 0.040 | 0.969 |
|  | rs174619 | 0.395 | 0.294 | 1.343 | 0.182 |  | 0.249 | 0.151 | 1.645 | 0.103 |  | -0.051 | 0.038 | -1.321 | 0.190 |  | -0.423 | 0.915 | -0.462 | 0.645 |
|  | rs498793 | -0.104 | 0.508 | -0.204 | 0.838 |  | 0.064 | 0.262 | 0.243 | 0.808 |  | 0.039 | 0.063 | 0.612 | 0.542 |  | -0.379 | 1.522 | -0.249 | 0.804 |
|  | rs3168072 | 0.264 | 0.333 | 0.793 | 0.429 |  | 0.098 | 0.172 | 0.568 | 0.571 |  | **-0.081** | **0.041** | **-1.990** | **0.049** |  | 0.596 | 1.036 | 0.576 | 0.566 |
| *Fads3* | rs174455 | 0.098 | 0.268 | 0.364 | 0.717 |  | 0.201 | 0.137 | 1.470 | 0.144 |  | -0.017 | 0.034 | -0.500 | 0.618 |  | -0.761 | 0.815 | -0.933 | 0.352 |
|  | rs174464 | 0.077 | 0.271 | 0.285 | 0.776 |  | 0.204 | 0.139 | 1.469 | 0.144 |  | -0.020 | 0.034 | -0.606 | 0.546 |  | 0.393 | 0.842 | 0.467 | 0.641 |
|  | rs76996928 | -0.229 | 0.249 | -0.921 | 0.359 |  | 0.151 | 0.128 | 1.180 | 0.241 |  | 0.003 | 0.032 | 0.102 | 0.919 |  | -0.996 | 0.757 | -1.317 | 0.190 |
| *Elovl2* | rs1323739 | -0.222 | 0.267 | -0.831 | 0.408 |  | -0.030 | 0.138 | -0.220 | 0.826 |  | 0.049 | 0.034 | 1.429 | 0.156 |  | -0.561 | 0.784 | -0.715 | 0.476 |
|  | rs2295602 | -0.089 | 0.419 | -0.213 | 0.831 |  | 0.203 | 0.215 | 0.945 | 0.347 |  | 0.046 | 0.057 | 0.818 | 0.415 |  | -0.372 | 1.266 | -0.294 | 0.769 |
|  | rs2180725 | 0.177 | 0.301 | 0.587 | 0.558 |  | 0.040 | 0.155 | 0.255 | 0.799 |  | 0.004 | 0.037 | 0.117 | 0.907 |  | 0.114 | 0.888 | 0.128 | 0.898 |
|  | rs3798710 | 0.087 | 0.252 | 0.346 | 0.730 |  | 0.017 | 0.130 | 0.133 | 0.895 |  | -0.046 | 0.033 | -1.396 | 0.166 |  | 0.287 | 0.750 | 0.383 | 0.702 |
|  | rs78793420 | -0.178 | 0.288 | -0.619 | 0.537 |  | -0.097 | 0.148 | -0.657 | 0.512 |  | 0.038 | 0.036 | 1.066 | 0.289 |  | -0.601 | 0.854 | -0.703 | 0.483 |
| *Elovl5* | rs209512 | 0.287 | 0.281 | 1.022 | 0.309 |  | 0.049 | 0.145 | 0.339 | 0.735 |  | -0.014 | 0.036 | -0.402 | 0.688 |  | 0.158 | 0.828 | 0.191 | 0.849 |
|  | rs2281274 | 0.063 | 0.305 | 0.207 | 0.836 |  | -0.050 | 0.157 | -0.322 | 0.748 |  | 0.032 | 0.037 | 0.864 | 0.390 |  | 0.167 | 0.895 | 0.187 | 0.852 |
|  | rs2294852 | -0.027 | 0.293 | -0.092 | 0.927 |  | -0.056 | 0.151 | -0.369 | 0.713 |  | -0.014 | 0.036 | -0.389 | 0.698 |  | 0.059 | 0.881 | 0.067 | 0.946 |
|  | rs2397142 | -0.105 | 0.297 | -0.354 | 0.724 |  | -0.050 | 0.153 | -0.329 | 0.743 |  | -0.018 | 0.036 | -0.499 | 0.619 |  | 0.648 | 0.878 | 0.738 | 0.462 |
|  | rs6909592 | -0.026 | 0.295 | -0.089 | 0.929 |  | -0.045 | 0.152 | -0.294 | 0.769 |  | -0.010 | 0.036 | -0.267 | 0.790 |  | 0.163 | 0.886 | 0.184 | 0.854 |
|  | rs9349665 | -0.166 | 0.303 | -0.548 | 0.585 |  | -0.039 | 0.156 | -0.252 | 0.801 |  | -0.010 | 0.037 | -0.481 | 0.631 |  | 0.273 | 0.892 | 0.306 | 0.760 |
|  | rs9395858 | -0.107 | 0.281 | -0.380 | 0.704 |  | 0.028 | 0.145 | 0.195 | 0.845 |  | 0.005 | 0.034 | 0.139 | 0.890 |  | 0.092 | 0.815 | 0.113 | 0.910 |
|  | rs12207094 | 0.104 | 0.403 | 0.259 | 0.796 |  | 0.026 | 0.208 | 0.127 | 0.899 |  | 0.041 | 0.050 | 0.832 | 0.407 |  | -0.416 | 1.213 | -0.343 | 0.732 |

Note: ALA: α-linolenic acid, EPA: eicosapentaenoic acid, DHA: docosahexaenoic acid, SNPs: Single nucleotide polymorphisms. *Fads*: fatty acid desaturases, *Elovl*: elongase of long chain fatty acid,

**Supplementary Table 6 Distributions of the 26 SNPs in the fatty acid desaturases and elongases of the subjects in different groups**

| Gene | SNPs | Call rate (%) | Genotype | High DHA-rich n-3 PUFAs intake group* (n=369) | Low DHA-rich n-3 PUFAs intake group^#^ (n=681) | *P* |
| --- | --- | --- | --- | --- | --- | --- |
| *Fads1* | rs174448 | 99.94 | AA/GG/AG | 243/14/112 | 452/25/204 | 0.984 |
|  | rs174537 | 99.97 | GG/TT/GT | 147/48/174 | 272/93/316 | 0.963 |
|  | rs174550 | 99.91 | CC/TT/CT | 54/142/173 | 101/267/313 | 0.960 |
|  | rs174553 | 99.89 | AA/GG/AG | 149/53/167 | 275/99/307 | 0.997 |
| *Fads2* | rs174598 | 99.18 | AA/TT/AT | 52/152/165 | 95/286/300 | 0.968 |
|  | rs174602 | 99.84 | CC/TT/CT | 22/203/144 | 45/373/263 | 0.919 |
|  | rs174609 | 99.89 | CC/TT/CT | 12/251/106 | 23/468/190 | 0.958 |
|  | rs174619 | 99.42 | AA/GG/AG | 23/220/126 | 39/405/237 | 0.935 |
|  | rs498793 | 100.00 | CC/TT/CT | 316/3/50 | 580/4/97 | 0.873 |
|  | rs3168072 | 99.91 | AA/TT/AT | 262/11/96 | 481/22/178 | 0.974 |
| *Fads3* | rs174455 | 100.00 | CC/TT/CT | 182/33/154 | 339/60/282 | 0.990 |
|  | rs174464 | 99.84 | AA/GG/AG | 21/233/115 | 41/431/209 | 0.969 |
|  | rs76996928 | 99.67 | CC/TT/CT | 85/114/170 | 154/213/314 | 0.985 |
| *Elovl2* | rs1323739 | 99.49 | CC/GG/CG | 34/165/170 | 65/306/310 | 0.977 |
|  | rs2295602 | 99.43 | CC/TT/CT | 310/4/55 | 568/7/106 | 0.958 |
|  | rs2180725 | 100.00 | CC/TT/CT | 24/213/132 | 42/400/239 | 0.944 |
|  | rs3798710 | 99.97 | CC/GG/CG | 71/122/176 | 134/222/325 | 0.981 |
|  | rs78793420 | 99.49 | CC/TT/CT | 19/211/139 | 36/392/253 | 0.984 |
| *Elovl5* | rs209512 | 100.00 | AA/GG/AG | 70/114/185 | 127/217/337 | 0.949 |
|  | rs2281274 | 99.84 | CC/TT/CT | 14/234/121 | 26/426/229 | 0.961 |
|  | rs2294852 | 99.75 | CC/GG/CG | 140/55/174 | 261/99/321 | 0.984 |
|  | rs2397142 | 99.82 | CC/GG/CG | 169/43/157 | 310/84/287 | 0.949 |
|  | rs6909592 | 99.94 | CC/GG/CG | 59/138/172 | 109/253/319 | 0.997 |
|  | rs9349665 | 100.00 | CC/TT/CT | 31/194/144 | 60/356/265 | 0.975 |
|  | rs9395858 | 99.86 | CC/TT/CT | 188/30/151 | 344/57/280 | 0.986 |
|  | rs12207094 | 100.00 | AA/TT/AT | 297/4/68 | 548/5/128 | 0.836 |

Note: *High DHA-rich n-3 PUFAs intake group was included maternal exogenous DHA-rich n-3 PUFAs supplementation at the early (S1) and middle (S2) pregnancy, ^#^Low DHA-rich n-3 PUFAs intake group was included maternal exogenous DHA-rich n-3 PUFAs supplementation at the late pregnancy (S3) and non-exogenous DHA-rich n-3 PUFAs supplementation during the whole pregnancy. SNPs: Single nucleotide polymorphisms, *Fads*: fatty acid desaturases, *Elovl*: elongase of long chain fatty acid, PUFAs: polyunsaturated fatty acids.
